# Identification of heptapeptides targeting a lethal bacterial strain in septic mice through an integrative approach

**DOI:** 10.1038/s41392-022-01035-6

**Published:** 2022-07-25

**Authors:** Xiaoyan Zhang, Shan Li, Haihua Luo, Shuyue He, Huangda Yang, Lei Li, Tian Tian, Qizheng Han, Jiacong Ye, Chenyang Huang, Aihua Liu, Yong Jiang

**Affiliations:** 1 Guangdong Provincial Key Laboratory of Proteomics, State Key Laboratory of Organ Failure Research, Department of Pathophysiology, School of Basic Medical Sciences, Southern Medical University, Guangzhou, 510515 China; 2 Department of Respiratory and Critical Care Medicine, Nanfang Hospital, Southern Medical University, Guangzhou, China

**Keywords:** Peptide delivery, Infection

## Abstract

Effectively killing pathogenic bacteria is key for the treatment of sepsis. Although various anti-infective drugs have been used for the treatment of sepsis, the therapeutic effect is largely limited by the lack of a specific bacterium-targeting delivery system. This study aimed to develop antibacterial peptides that specifically target pathogenic bacteria for the treatment of sepsis. The lethal bacterial strain *Escherichia coli MSI001* was isolated from mice of a cecal ligation and puncture (CLP) model and was used as a target to screen bacterial binding heptapeptides through an integrative bioinformatics approach based on phage display technology and high-throughput sequencing (HTS). Heptapeptides binding to E. coli *MSI001* with high affinity were acquired after normalization by the heptapeptide frequency of the library. A representative heptapeptide VTKLGSL (VTK) was selected for fusion with the antibacterial peptide LL-37 to construct the specific-targeting antibacterial peptide VTK-LL37. We found that, in comparison with LL37, VTK-LL37 showed prominent bacteriostatic activity and an inhibitive effect on biofilm formation in vitro. In vivo experiments demonstrated that VTK-LL37 significantly inhibited bacterial growth, reduced HMGB1 expression, alleviated lesions of vital organs and improved the survival of mice subjected to CLP modeling. Furthermore, membrane DEGP and DEGQ were identified as VTK-binding proteins by proteomic methods. This study provides a novel strategy for targeted pathogen killing, which is helpful for the treatment of sepsis in the era of precise medicine.

## Introduction

Sepsis is a systemic inflammatory response syndrome caused by pathogen infection^1,2^ and can further develop into septic shock, potentially leading to multiple organ failure (MOF). Currently, sepsis has become the leading cause of the mortality of patients in intensive care units (ICUs).^3–5^ The hospital mortality of sepsis can reach 15–50%,^6^ which becomes a harmful threat to human health and leads to a heavy social and financial burden worldwide.^7–9^ Identifying effective approaches to control sepsis has become an important goal for biological scientists and medical doctors in the clinic.^10^

Pathogen infection is the fundamental cause of sepsis.^11^ Early and effective inhibition or killing of pathogens is essential for the prevention and treatment of sepsis.^12^ It is well established that different pathogens have different sensitivities to antibacterial drugs, characteristics of organ distribution and other biological behaviors, which lead to different clinical manifestations and prognoses.^13,14^ Therefore, it is urgent to establish an individualized medical strategy targeting specific pathogens for the treatment of sepsis. At present, various commercially available antimicrobial drugs exhibit some prominent defects, including nonspecific targeting and low concentrations in the lesion, which leads to low efficiency, high toxicity, various side effects^15^ and multidrug resistance.^16,17^ In the era of precision medicine, targeted drug design has been intensively developed,^18^ and high efficiency and sufficient safety have become the main goals for targeted drug design.^19^ Therefore, it is important to develop a targeted antimicrobial drug delivery system as a novel strategy for the prevention and treatment of sepsis.

The phage display technique was originally introduced as an efficient screening system in 1985.^20^ As a potent tool for the screening of binding partners, phage display has been widely applied in the discovery of blood-brain barrier (BBB) shuttle peptides,^21^ the identification of antibodies,^22,23^ antigen epitope screening,^24,25^ the development of new vaccines and drugs^26–28^ and the characterization of peptides for drug delivery.^29,30^ Following the development of next-generation sequencing (NGS) and bioinformatics technology, there is a promising opportunity for the application of phage display techniques to obtain high-affinity peptides for the construction of “biological missiles” targeting bacteria.

As a class of small bioactive molecular peptides, antibacterial peptides are produced by the immune defense system of organisms, including the human body. The antibacterial peptide LL-37 from humans was found to have broad-spectrum antibacterial activity^31–34^ by increasing the membrane permeability of bacteria, neutralizing endotoxins, and suppressing the production of proinflammatory factors.^35^ For this reason, LL-37 is considered an ideal template or a molecular skeleton for the development of new antimicrobial drugs.^36^

To develop a systematic approach for the targeted antibacterial therapy of sepsis, in this study, we isolated a new strain of lethal bacterium with genetic identification from septic mice and acquired heptapeptides with high affinity to the bacterium with a systematic approach incorporating the phage display technique, high-throughput sequencing (HTS) and bioinformatics analysis. High-affinity heptapeptides binding to the lethal bacterium were fused with LL37 to obtain functional hybrid peptides, and their antibacterial activities were evaluated both in vitro and in vivo. Our study provides a novel strategy for the systematic solution to the precise treatment of sepsis.

## Results

### Identification of the pathogenic bacterial strains from CLP mice

**Fig. 1:**
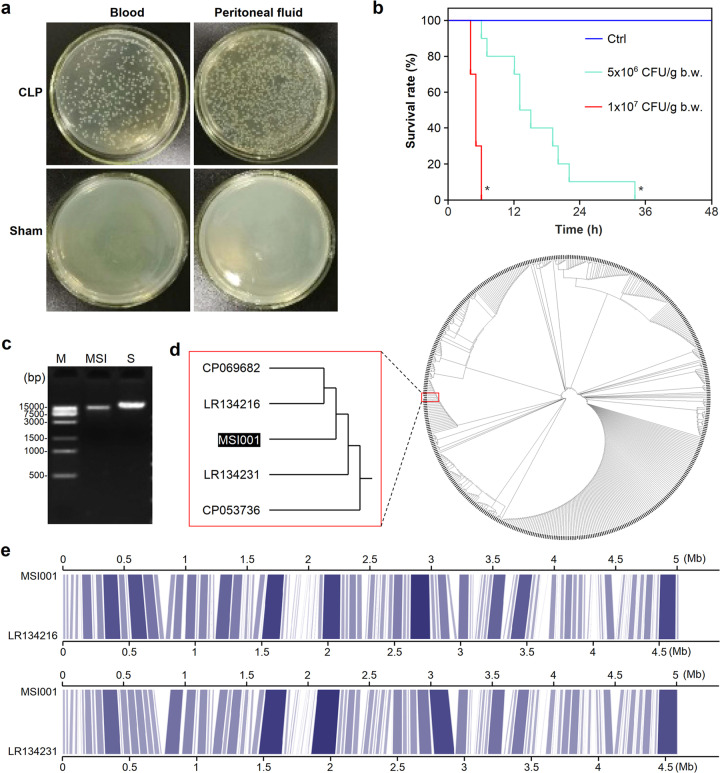
Identification of a pathogenic bacterial strain from CLP mice. **a** Bacterial cultures with blood and peritoneal fluid from C57 mice subjected to CLP modeling. Both blood and peritoneal fluid were acquired from C57 mice subjected to CLP modeling or wild-type C57 mice as controls. Bacterial culture was performed on agar plates at 37 °C overnight. **b** Survival analysis of mice challenged with the pathogenic bacterium. C57 mice were injected with bacteria at 5 × 10^6^ CFU/g body weight (low-dose group, green line) or 1 × 10^7^ CFU/g body weight (high-dose group, red line. Control mice received injection of an equivalent volume of normal saline (NS). The survival of mice was observed for 48 h after peritoneal injection, and Kaplan-Meier log-rank analysis was applied to test the significance of the difference in survival between the CLP mice and the control mice. (*n* = 10). *P < 0.05, compared to the control. **c** Agarose gel electrophoretogram of genomic DNA from the bacterial strain *MSI001*. The genomic DNA extracted from bacteria was resolved by electrophoresis on a 1% agarose gel. M: DNA marker; MSI: genomic DNA extracted from the bacterial strain *MSI001*; S: DNA standard. **d** Phylogenetic tree for genomic DNA of the bacterial strain *MSI001* isolated from CLP mice. The genomic DNA sequence of strain *MSI001* was blasted against the nucleotide (nt) sequence database using BLAST^+^ (v2.11.0) (https://ftp.ncbi.nlm.nih.gov/blast/executables/blast+/LATEST/). Marker genes were identified by using the Genome Taxonomy Database Toolkit (GTDB-Tk) as described in the Methods, and the result was input into IQ-TREE for phylogenetic inference. The Interactive Tree of Life (iTOL) was used to plot the evolution distance tree. **e** Visualization of whole-genome alignment (WGA) of *MSI001* with the two closest reference genomic DNA samples from E. coli LR134216 (upper panel) and LR134231 (lower panel). The alignment result was visualized by Kablammo (http://kablammo.wasmuthlab.org/) with parameter settings of bit score > 10,000 and E value < 1E-10. In the figure, the blue lines represent the homology between the *MSI001* sequence and the reference sequence. The dark intensity of the blue lines indicates the value of the bit score. The white interval in the genomic DNA of *MSI001* and the E. coli strains (LR134216 and LR134231) indicates that the target sequence failed to align with the reference sequence for low bit scores or insignificant E values

To acquire bacterial strains that induce septic infection, we reproduced a cecal ligation and puncture (CLP) model in mice. Samples collected from either blood or peritoneal fluid from CLP mice, but not sham mice, led to bacterial colony growth on agar plates (Fig. 1a). To investigate whether the bacterial strains isolated from the CLP mice were pathogenic, we analyzed the mortality of the mice subjected to peritoneal injection of bacteria and found that some bacterial strains were of high mortality for the mice challenged with bacterial injection (Supplementary Fig. S1). The bacterial strain with the highest death rate was chosen for further analysis of the survival of mice. The 24 h survival rate of mice challenged with low-dose (5 × 10^6^ CFU/g) pathogenic bacteria was 10%, and there was no survival for the mice subjected to high-dose (1 × 10^7^ CFU/g) injection of this strain, which was significantly lower than that of the control group (*P* < 0.05) (Fig. 1b). This result indicated that this bacterial strain isolated from the blood of CLP mice was lethal and suitable for the reproduction of sepsis. Based on the capability to induce sepsis in mice, we named this strain mouse sepsis inducer 001 (*MSI001*, GenBank accession number: CP076645).

To further identify the bacterial strain, bacterial genomic DNA was extracted for HTS (Fig. 1c). We found that the read length of DNA sequencing was approximately 10,000 bp, and the read quality had a peak greater than 0.9 (Supplementary Fig. S2). The BLAST results showed that the lethal strain isolated from the blood of CLP mice belonged to the species *Escherichia coli* (E. coli) with a neighborhood of LR134216 and LR134231 in evolution (Fig. 1d). Whole-genome alignment (WGA) visualization showed that the *MSI001* strain was highly homologous to E. coli strains LR134216 and LR134231 in genomic evolution (Fig. 1e).

### Screening and sequencing of phages specifically binding to the lethal bacterium

**Fig. 2:**
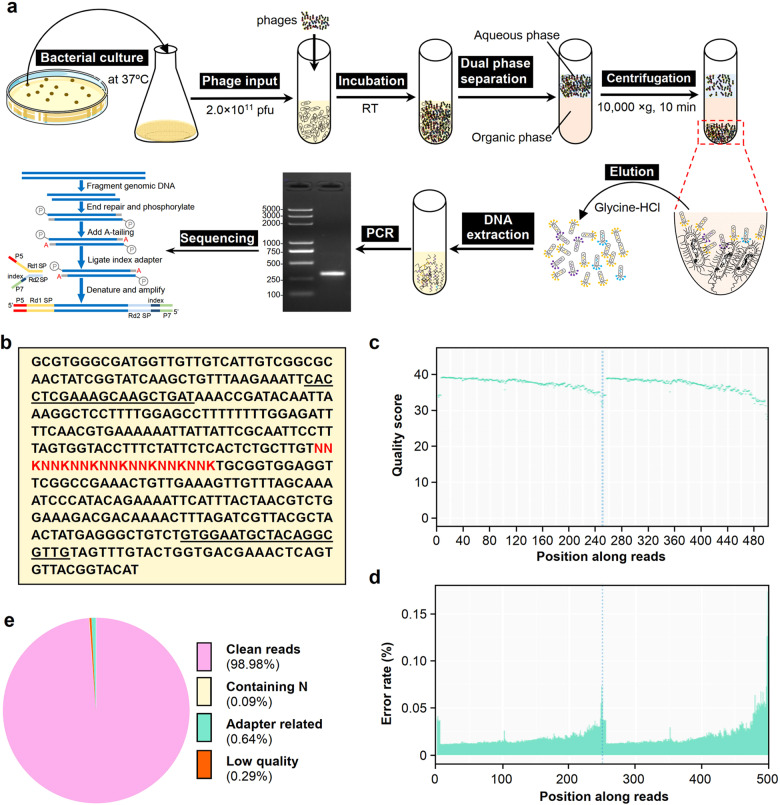
Biopanning and sequencing of phages specifically binding to pathogenic bacteria. **a** Flowchart for biopanning of heptapeptides (C7Cs) that bind to pathogenic bacteria from a phage display random peptide library. After being cultured at 37 °C to an OD_600_ of approximately 1.0, the bacteria were collected and incubated with 2 × 10^11^ pfu phages in a final volume of 250 μL at room temperature for 10 min. The mixture was separated by the modified Biopanning and Rapid Analysis of Selective Interactive Ligands (BRASIL) method. After centrifugation at 10,000 × g for 10 min, the precipitated phages bound to bacteria were washed 3 times with PBS. The phages were eluted with glycine-HCl, followed by DNA extraction and PCR amplification. The PCR products containing the sequences encoding heptapeptides were resolved by electrophoresis on 2% agarose and collected for HTS. **b** The DNA sequence encoding random C7C peptides and protein III fusion protein (C7C-PIII). The NNK sequence (red) represents the DNA sequences for random heptapeptides fused to the sequence of phage gene III. The primers designed as the underlined sequences were used to amplify the DNA encoding heptapeptides. Here, N represents any nucleobase, G, C, A or T, while K represents G or T. **c** Quality score distribution along reads of PCR products of DNA encoding heptapeptides. The PCR products were sequenced by using a MiSeq instrument (Illumina) with a 250-bp paired-end-read format. **d** Error rate distribution along reads. The PCR products were sequenced as described in (**c**). **e** The composition of the raw data from HTS. The reads, including clean reads, reads containing N, adapter-related reads and low-quality sequences, were analyzed for the quality control of sequencing

To acquire peptides targeting the pathogenic bacterium, phage display was performed to screen C7C peptides with specific binding activity. The flow chart for phage biopanning and DNA sequencing for heptapeptide-specific binding to lethal bacteria is shown in Fig. 2a. The DNAs encoding random C7C peptides were fused with the sequence for protein III (PIII) to form gene sequences encoding C7C-PIII fusion proteins (Fig. 2b). The primers corresponding to the underlined sequences in Fig. 2b were used to amplify the DNA encoding heptapeptides. PCR products of phage DNAs were subjected to HTS.

The sequencing data for the phage library or sublibraries of phages binding to the pathogenic bacteria were obtained with a volume of 46.63 G and 4.45 G, respectively (Supplementary Table S1). Sequencing quality along reads was distributed in Q30 to Q40, with an accuracy higher than 99.9% (Fig. 2c), and the error rate for most reads was below 0.025% (Fig. 2d). Generally, the sequencing data were composed of 98.98% clean reads, 0.09% containing N reads, 0.64% adapter-related reads and 0.29% low-quality reads (Fig. 2e). The results showed that high-quality sequencing data had been acquired.

### Normalization of heptapeptide binding to the lethal E. coli strain *MSI001*

The C7C phage library has a diversity of 1.28 × 10^9^, which covers almost all possible combinations of amino acids (AAs). Due to the bias in the composition of phages, different heptapeptides encoded by these phages were found with different frequencies in the phage library. For example, the frequencies of GSAPVRS and LTAKHMQ were 7,741 and 7,257, respectively (Supplementary Table S2), whereas the lowest frequency was only 1, indicating that the frequency of heptapeptide distribution was extremely heterogenous. Thus, calibration was required to reduce the bias effect of the phage library.

**Fig. 3:**
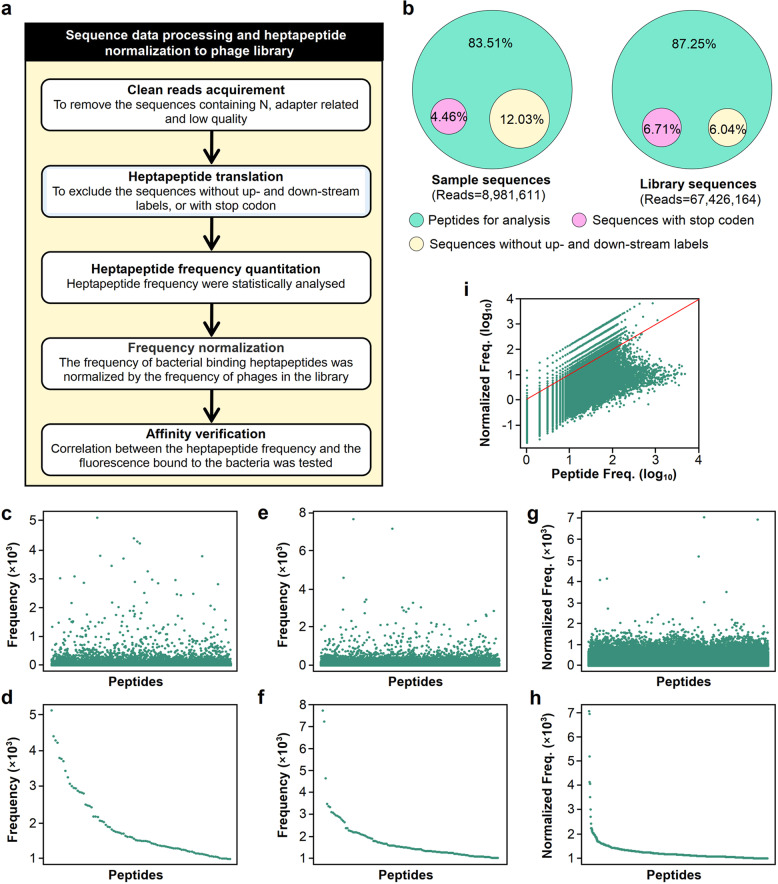
Frequency calibration for heptapeptides binding to the lethal bacterial strain *MSI001*. **a** Flowchart for sequence data processing and heptapeptide normalization to the phage library. **b** Sequence composition of recovered samples and phage library inputs. Peptides for analysis were acquired by removing the sequences with stop codons or without up- and/or down-stream labels. **c** Randomized frequency distribution for the binding heptapeptides. **d** Descending frequency distribution for the binding heptapeptides with frequencies above 1000. **e** Randomized frequency distribution for the heptapeptides encoded by the phage library. **f** Descending frequency distribution for the library heptapeptides with frequencies above 1000. **g** Normalized frequency distribution for binding heptapeptides. The frequency of each binding heptapeptide was divided by the frequency of the corresponding heptapeptide encoded by the phage library to acquire the normalized frequency of binding heptapeptides. **h** Descending frequency distribution of binding heptapeptides with normalized frequency higher than 1000. **i** Logarithmic distribution of unnormalized and normalized heptapeptide frequencies. The frequency of all peptides except those on the red line were adjusted after normalization

The flowchart for the process of DNA sequencing and heptapeptide normalization of the phage library is shown in Fig. 3a. The sequences with stop codons or without up- and/or downstream labels were removed, and clean sequence data were acquired from the samples or phage library with overall percentages of 83.51% and 87.25%, respectively (Fig. 3b). There were 27 heptapeptides binding to pathogenic bacteria with a frequency higher than 2,000, and the highest frequency was 5,124 (Fig. 3c, d; Supplementary Table S3). The frequencies of binding heptapeptides were normalized by the frequency of a corresponding peptide in the phage library, resulting in a substantial adjustment to the ranking of the binding heptapeptides. For example, the peptide IHSPTAL was originally ranked at position 1 but ranked 564,599 after normalization against the library (Supplementary Table S4); in contrast, the peptide QTYHSGH ranked 106 before normalization, whereas its ranking was elevated to position 1 after normalization against the library (Supplementary Table S5). In fact, frequency normalization notably changed the overall distribution profiles of binding heptapeptides (Fig. 3g, h). The frequencies of the binding heptapeptides except those along the red line were substantially adjusted (Fig. 3i).

### Physical features of heptapeptides binding to lethal E. coli *MSI001*

**Fig. 4:**
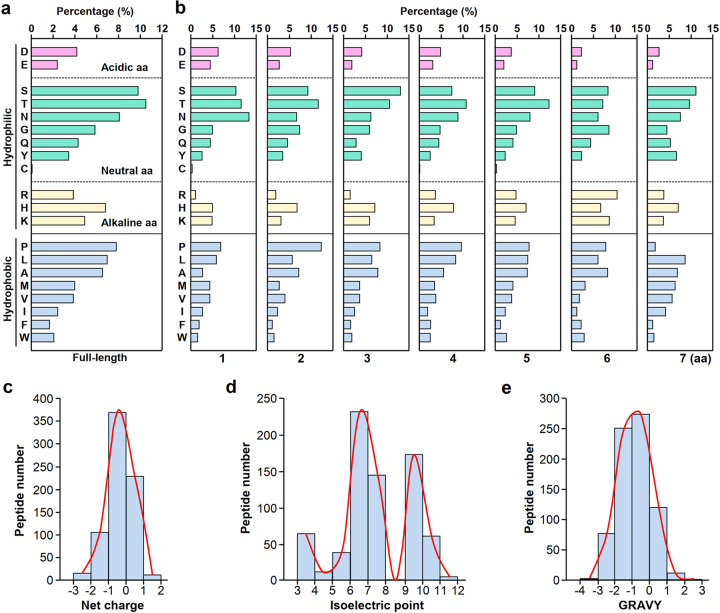
Physical characteristics of the heptapeptides binding to the lethal bacterial strain *MSI001*. **a** Amino acid (AA) composition of the bacterial binding heptapeptides. According to the physical properties, all 20 AAs were divided into hydrophilic and hydrophobic AAs. There are three kinds of hydrophilic AAs, including 2 acidic AAs, i.e., aspartic acid (D) and glutamic acid (E); 7 neutral AAs, i.e., serine (S), threonine (T), asparagine (N), glycine (G), glutamine (Q), tyrosine (Y) and cystine (C); and 3 alkaline AAs, i.e., arginine (R), histidine (H) and lysine (K). The hydrophilic AAs were proline (P), leucine (L), alanine (A), methionine (M), valine (V), isoleucine (I), phenylalanine (F) and tryptophan (W). **b** AA composition of the bacterial binding heptapeptides in different positions (AAs 1-7) of the 7-mer peptides. **c** Statistical analysis of the electrical properties of the bacterial binding heptapeptides. The net charge of each heptapeptide was acquired by online Protein Calculator v3.4 software (http://protcalc.sourceforge.net). **d** Compositional analysis of the isoelectric point (PI) of the bacterial binding heptapeptides. The PI value of each heptapeptide was acquired by online Protein Isoelectric Point Calculator software (http://www.endmemo.com/bio/proie.php). **e** The hydropathicity distribution of the bacterial binding heptapeptides. The GRAVY value was predicted online at http://www.gravy-calculator.de/index.php

The binding heptapeptides were composed of hydrophilic and hydrophobic AAs. There are three kinds of hydrophilic AAs, i.e., acidic AAs, neutral AAs and alkaline AAs (Fig. 4a). Overall, the most abundant AA residue was threonine (T), and the rarest was cysteine (C). At AAs 1 to 7 across the heptapeptides, asparagine (N), proline (P), serine (S), threonine (T), threonine (T), arginine (R) and serine (S) were the most abundant residues (Fig. 4b).

To further describe the physical features of the peptides, we analyzed the net charge, isoelectric point (PI) and hydropathicity of the binding heptapeptides with online software. We found that the net charge distribution of the binding heptapeptides was between −3 and 2 with a peak between −1 and 0 (Fig. 4c), their PI was distributed between 3 and 12 with an outline of double peaks (Fig. 4d), and the grand average of hydropathicity (GRAVY) value distribution was between −4 and 3 with an apex between −1 and 0 (Fig. 4e).

### Validation of binding affinity between the heptapeptides and lethal E. coli *MSI001*

**Fig. 5:**
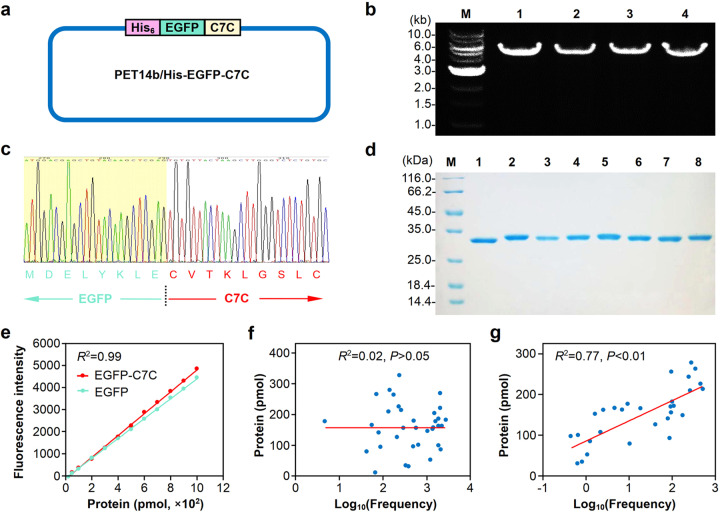
Validation of binding affinity between the heptapeptides and the lethal bacterial strain *MSI001*. **a** Construction of the expression vector for the fusion protein of His-tagged EGFP and heptapeptides. The heptapeptide-encoding DNA sequence was amplified by PCR and inserted into the expression vector pET14b/His-EGFP to construct the recombinant plasmid expressing His-EGFP-C7C. **b** Electrophoretogram of PCR products for the construction of plasmids expressing His-tagged EGFP- or EGFP-fused heptapeptides. The PCR products were resolved by electrophoresis on a 1% agarose gel. M represents the DNA marker; lane 1 is the PCR product for pET14b/His-EGFP; lanes 2-4 are PCR products for pET14b/His-EGFP-C7C. **c** Identification of plasmids expressing His-EGFP-C7C fusion proteins by DNA sequencing. The DNA sequence for EGFP is indicated with a green arrow, and that for heptapeptide is labeled with a red arrow. **d** SDS-PAGE of His-tagged EGFP fusion proteins. Purified fusion proteins, including His-EGFP or His-EGFP-C7C, were resolved by 12% SDS-PAGE. M represents the protein marker; lane 1 is the His-tagged EGFP fusion protein; lanes 2-8 are the His-EGFP-C7C fusion proteins. **e** Standard curves for fluorescence intensity and His-tagged EGFP fusion proteins. The fluorescence intensity was quantitated by spectrophotometry. Formulas were constructed to calculate the amount of His-EGFP or His-EGFP-C7C fusion proteins. **f** The correlation between heptapeptide frequency and the fluorescence intensity of the corresponding His-EGFP-C7C fusion proteins. The bacteria were incubated with the His-EGFP-C7C fusion proteins at 25 °C for 4 h, and the fluorescence of the binding heptapeptides was quantitated by spectrophotometry. The correlation between the log-transformed heptapeptide frequency and the fluorescence of the corresponding His-EGFP-C7C was analyzed with SPSS (v22). **g** The correlation between normalized heptapeptide frequency and the fluorescence intensity of the corresponding His-EGFP-C7C fusion proteins. The binding process and fluorescence quantitation are described in (**f**). The correlation between the normalized heptapeptide frequency with log transformation and the fluorescence of the corresponding His-EGFP-C7C was analyzed

To validate the relationship between the heptapeptide frequency and the binding affinity to E. coli *MSI001*, DNA sequences encoding heptapeptides were fused to the EGFP sequence to construct His-EGFP-C7C-expressing vectors (Fig. 5a). Linearized pET14b with insertion of DNAs encoding His-EGFP-C7C was amplified by PCR (Fig. 5b; Supplementary Table S6), and the recombinant plasmids were identified by DNA sequencing (Fig. 5c). Then, the verified plasmids were transformed into E. coli BL21(DE_3_) for the expression of His-tagged recombinant proteins, and the fusion proteins His-EGFP-C7C and His-EGFP were successfully expressed and purified (Fig. 5d).

Standard curves of the fluorescence intensity of EGFP-fusion proteins were constructed for protein quantitation (Fig. 5e). The correlation between heptapeptide frequency and the fluorescence intensity of His-EGFP-C7C was analyzed, and we found that the log-transformed heptapeptide frequency without normalization and the fluorescence intensity of His-EGFP-C7C failed to show any significant correlation (*R*² = 0.02, *P* > 0.05) (Fig. 5f). Intriguingly, normalization of heptapeptide frequency against the library brought about a significant correlation (*R*² = 0.77, *P* < 0.01) between log-transformed frequency and the fluorescence intensity of His-EGFP-C7C (Fig. 5g).

### Antimicrobial activity of the C7C-LL37 in vitro

**Fig. 6:**
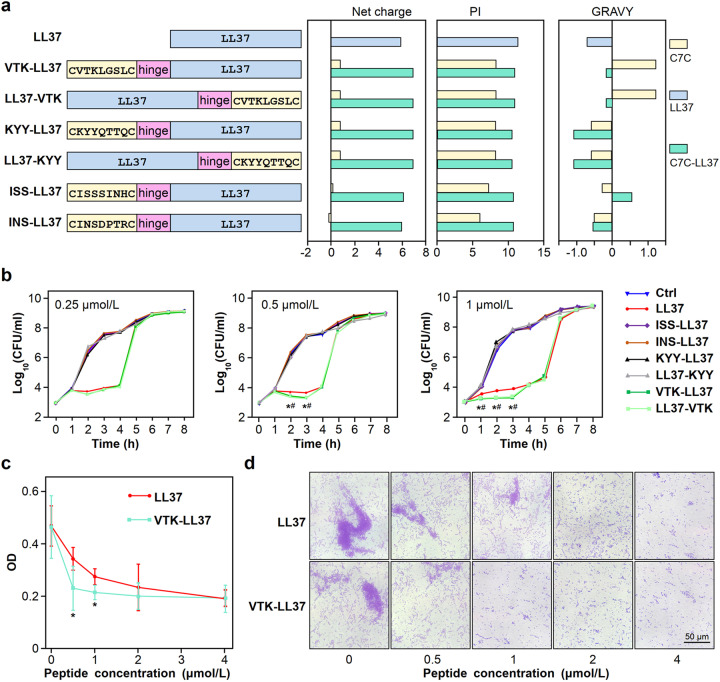
Antimicrobial activity of the C7C-LL37 fusion peptides in vitro. **a** Primary structures and physical properties of the C7C and LL37 fusion peptides, including LL37, VTK-LL37, LL37-VTK, KYY-LL37 and LL37-KYY, ISS-LL37, INS-LL37. Net charge, PI and GRAVY values were acquired as described above. **b** Inhibitory effect of C7C-LL37 fusion peptides on bacterial growth. A C7C-LL37 fusion peptide or LL37 alone was added to the cultured bacterial strain *MSI001* (1 × 10^3^ CFU) at final concentrations of 0.25 μmol/L, 0.5 μmol/L and 1 μmol/L. Sterile DPBS was used as control. After incubation with the C7C-LL37 or LL37 peptides at 37 °C for different times (1-8 h), bacterial colonies were counted. *n* = 3. *P < 0.05, VTK-LL37 group compared to LL37 group; ^#^*P* < 0.05, LL37-VTK group compared to LL37 group. **c**, **d** Inhibitory effect of VTK-LL37 on biofilm formation. The VTK-LL37 or LL37 peptide was added to the bacterial culture at a final concentration of 0-4 μmol/L and incubated at 37 °C for 3 days. Bacterial culture medium and the peptides were refreshed every day. The biofilm was stained with 1% crystal violet, and the precipitates were dissolved in 95% ethanol, followed by detection of absorbance at 570 nm (A_570_) (**c**). Moreover, the biofilm was observed under a microscope (**d**). *n* = 5. *P < 0.05, VTK-LL37 group compared to LL37 group

Several high-affinity heptapeptides, including VTKLGSL (VTK), KYYQTTQ (KYY), ISSSINH (ISS) and INSDPTR (INS), were used to synthesize C7C-LL37 fusion peptides, including VTK-LL37, LL37-VTK, KYY-LL37, LL37-KYY, ISS-LL37 and INS-LL37 (Fig. 6a). The physical features of the high-affinity heptapeptides and C7C-LL37 fusion peptides were analyzed (Fig. 6a; Supplementary Table S7).

Importantly, we found that both VTK-LL37 and LL37-VTK not only inhibited *MSI001* bacterial growth but also showed stronger antibiotic activity than LL37 alone in the early stage (1-3 h) of bacterial growth (Fig. 6b). Moreover, we also found that VTK-LL37 inhibited biofilm formation of E. coli *MSI001*, and VTK-LL37 had a stronger inhibition of biofilm formation at a low dose than LL37 alone (Fig. 6c, d).

In order to clarify the antibacterial activity of VTK-LL37 on the drug-resistant strains, we performed an experiment with the *MSI001* strain transformed with a plasmid containing an *Amp*^*+*^ gene, and found that VTK-LL37 had a more significant inhibitive effect on the bacterial growth of the ampicillin-resistant *MSI001* bacteria in comparison with LL37 (Supplementary Fig. S4a).

### Therapeutic effect of VTK-LL37 in vivo

**Fig. 7:**
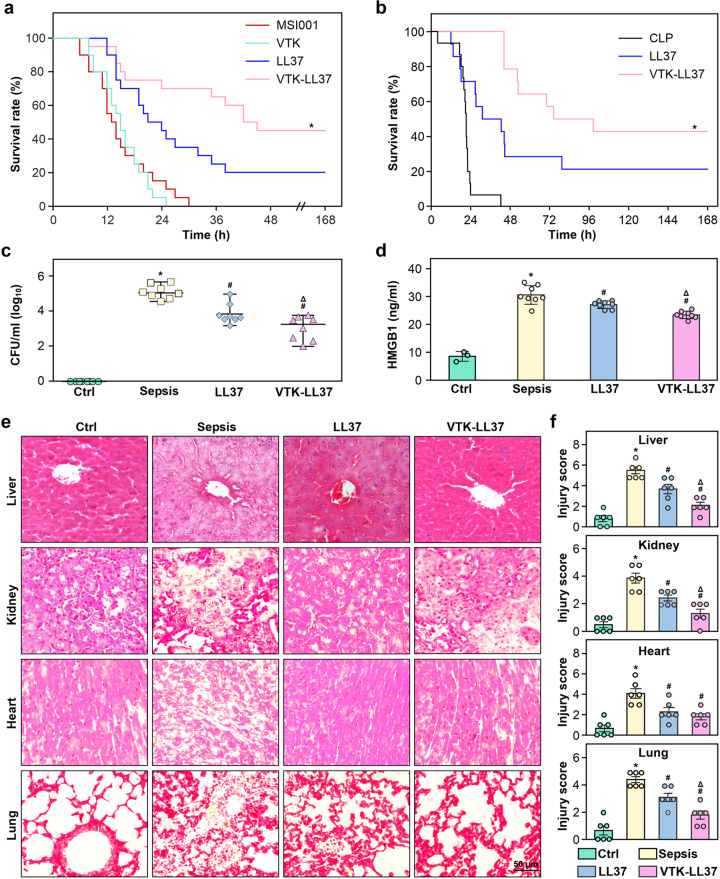
The therapeutic effect of the bacterial binding heptapeptide VTK and LL37 fusion peptide VTK-LL37 on septic mice. **a** The effect of VTK-LL37 on the survival of mice subjected to septic modeling with the bacterial strain *MSI001*. Wild-type C57 mice were intraperitoneally injected with *MSI001* bacteria (6 × 10^6^ CFU/g body weight) to reproduce a sepsis model. One hour later, the mice were intravenously injected with VTK, LL37 or VTK-LL37 peptide at 0.2 nmol/g body weight. Normal saline (NS) was used as control. The mice were observed for survival up to 168 h after bacterial injection, and Kaplan-Meier log-rank analysis was performed to test the significance of differences between groups. *n* = 20. *P < 0.05, compared to LL37 group. **b** Effect of VTK-LL37 and LL37 on the survival of CLP mice. One hour after CLP modeling, the mice were intravenously injected with LL37 or VTK-LL37 peptide at a dosage of 0.2 nmol/g body weight. Equal volume of normal saline (NS) was used as control. The mice were observed for survival up to 168 h after CLP modeling, and Kaplan-Meier log-rank analysis was performed to test the significance of differences between groups. *n* = 15; *P < 0.05, compared to LL37 group. **c** Inhibitory effect of VTK-LL37 on bacterial growth in the blood of mice subjected to sepsis modeling. The mice were intraperitoneally injected with *MSI001* bacteria. One hour later, the mice were subjected to tail vein injection (TVI) with VTK-LL37 (VTK-LL37 group), LL37 (LL37 group) or an equivalent volume of NS (sepsis group). Wild-type mice without modeling were used as controls (control group). The blood bacteria were quantitated as colony forming units (CFU) 12 h after injection of bacteria. *n* = 8. *P < 0.05, compared to control; ^#^*P* < 0.05, compared to sepsis group^; Δ^*P* < 0.05, compared to LL37 group. Results are expressed as the median with range. **d** Suppression of VTK-LL37 on the production of HMGB1 in the blood of septic mice. One hour after intraperitoneal injection of bacteria, the mice were subjected to TVI with VTK-LL37, LL37 or equivalent NS. Twelve hours after bacterial injection, HMGB1 in the serum was quantitated by ELISA. *n* = 8. *P < 0.05, compared to control; ^#^*P* < 0.05, compared to sepsis group; ^Δ^*P* < 0.05, compared to LL37 group^.^ Results are expressed as the mean ± SD. **e**, **f** Protective effect of VTK-LL37 against organ damage in mice subjected to bacterial injection. One hour after sepsis modeling, mice were intravenously injected with VTK-LL37 peptide or an equivalent volume of NS. The liver, kidney, heart and lung tissues were collected 12 h after bacterial injection, followed by routine preparation for pathological examination. Hematoxylin and eosin (HE) staining was performed for histomorphological evaluation by microscopy. The injury scores of liver, kidney, heart and lung were calculated according to the criteria described in the Methods. *n* = 6. *P < 0.05, compared to control group; ^#^*P* < 0.05, compared to sepsis group; ^Δ^*P* < 0.05, compared to LL37 group

The survival of mice subjected to septic modeling with E. coli *MSI001* was recorded to assess the therapeutic effect of VTK-LL37 on sepsis. Kaplan-Meier survival analysis showed that VTK-LL37 significantly increased the survival of septic mice (Fig. 7a).

To determine the effect of VTK-LL37 on polymicrobial sepsis, we performed experiment to assess the therapeutic effect of VTK-LL37 on the survival of CLP mice. Kaplan-Meier survival analysis showed that VTK-LL37 also significantly increased the survival of sepsis mice induced by CLP modeling (Fig. 7b), which showed a 24 h survival rate similar to the mice subjected to E. coli *MSI001* injection (Supplementary Fig. S5).

Bacteremia is considered to be a key factor for the diagnosis of sepsis. To evaluate the antimicrobial activity of VTK-LL37 in vivo, we drew blood from mice for bacterial culture and found that VTK-LL37 had the stronger capability to suppress the bacterial growth of E. coli *MSI001* in the blood of septic mice than LL37 (Fig. 7c). HMGB1 is involved in the development of the systemic inflammatory response and plays a key role in the progression of sepsis. We found that VTK-LL37 significantly suppressed the production of HMGB1 in the serum of septic mice, which was superior to LL37 (Fig. 7d). We further evaluated the efficacy of VTK-LL37 on organ injury induced by sepsis and found that VTK-LL37 alleviated sepsis-induced injury to multiple organs, including the liver, lung, kidney and heart (Fig. 7e, f).

In addition, we performed experiment to clarify the safety of VTK-LL37 in vivo, and found that there were no perceivable side-effects on the vital organs, including heart, liver, kidney and lung, of mice even treated with 10× therapeutic dosage of VTK-LL37 (Supplementary Fig. S6).

### Identification of VTK-binding proteins on the membrane of E. coli *MSI001*

**Fig. 8:**
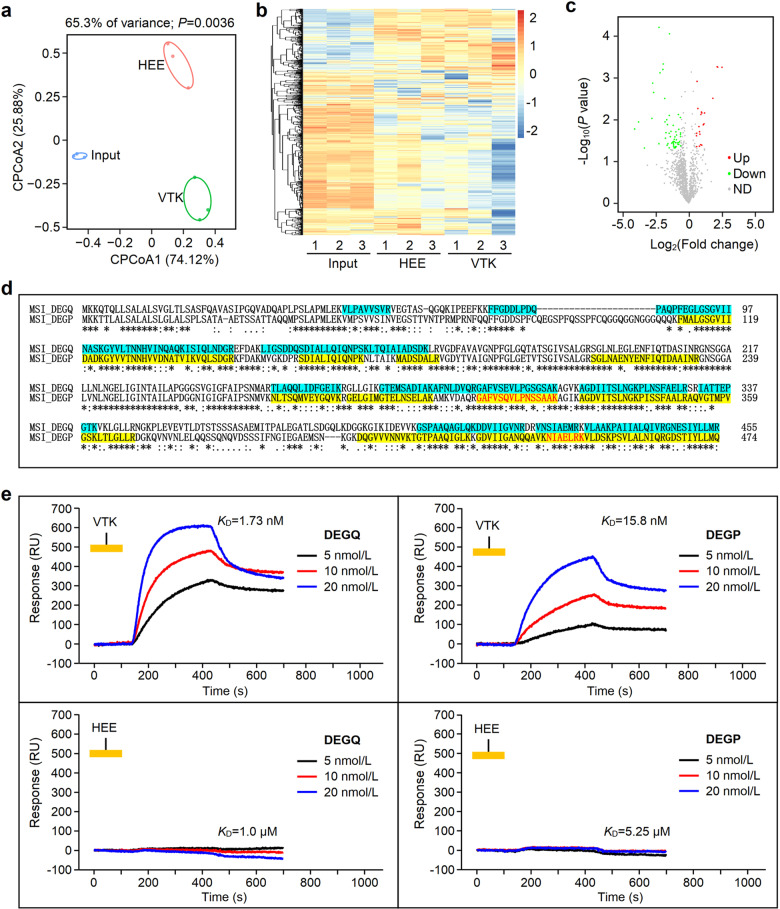
Identification of VTK heptapeptide-targeted bacterial membrane proteins with label-free mass spectrometry. **a** Constrained principal coordinates analysis (cPCoA) plot. Protein profiles were determined by cPCoA distance among samples and characterized by close intragroup distances among samples. **b** Hierarchical clustering analysis of proteins from the HEE, VTK and input groups. The outer membrane proteins of *MSI001* bacteria were digested with trypsin and pulled down by VTK or HEE heptapeptide as described in the Methods. Label-free mass spectrometry was used to quantitate proteins from the HEE, VTK and input groups. Red represents high expression, while blue represents low expression. **c** Volcano plot of proteins from the VTK group and HEE group. Red and green dots represent up- and downregulated proteins with |fold change (FC) | > 1.5 and *P*-value less than 0.05 compared to the control, respectively. **d** Protein sequence aliment of DEGQ and DEGP of E. coli *MSI001*. The AAs with background color represent peptide fragments identified by mass spectrometry. **e** SPRi assay for the binding affinity between VTK peptide and DEGP or DEGQ protein. The synthesized VTK peptide (5 mmol/L, 10 µL) or HEE peptide as control was immobilized on an SPRi chip. DEGP or DEGQ protein at different concentrations (5 nmol/L, 10 nmol/L, 20 nmol/L) in solution flowed on an SPRi chip. Binding data were collected and analyzed by commercial SPRi analysis software (Plexera SPR Data Analysis Model, Plexera, USA). The *K*_D_ values were obtained via the BIAevaluation Software version 4.1

To identify the bacterial membrane proteins binding with VTK heptapeptide, we used biotin-labeled VTK to perform pull-down assays. Constrained principal coordinates analysis (cPCoA) was performed to test the intragroup reproducibility and difference between groups, and found that the intragroup reproducibility was satisfied and that the difference between groups was significant (Fig. 8a). We further performed clustering analysis to assess the relationship of proteins interacting with the heptapeptide VTK and found that in comparison with the control group, VTK-interacting proteins indeed displayed a different clustering profile (Fig. 8b).

Volcano plots were generated to display the different proteins pulled down by the VTK or HEE peptide from the membrane proteins of E. coli *MSI001*. Compared to the control, the proteins with a *P*-value < 0.05 and |fold change (FC) | > 1.5 were considered to be significantly different. We found 21 upregulated proteins and 57 downregulated proteins in the VKT group (Fig. 8c). To evaluate the protein enrichment by pull-down assay, additional volcano plots of the HEE or VTK groups were also generated against the input as background, and we found that 112 and 131 proteins were significantly upregulated in the HEE and VTK groups, respectively (Supplementary Fig. S7a, b). In comparison with the input, the identified proteins with a *P*-value<0.05 were considered differential proteins. A Venn diagram was created to show the distribution of differential and coincidental proteins in different groups (Supplementary Fig. S7c).

Interestingly, we found that DEGQ and DEGP were highly enriched in comparison with either the input or HEE groups. The results of sequence alignment in the UniProt database showed that DEGQ and DEGP were highly homologous, and the identity of both proteins in the alignment is illustrated in Fig. 8d. The protein fragments identified by mass spectrometry (MS) were labeled with different colors (Fig. 8d). Some peptide fragments, such as GAFVSQVLPNSSAAK and NIAELRK from DEGP, were identified with high binding activity to VTK heptapeptide (Supplementary Fig. S8).

To determine the binding affinity of DEGP/DEGQ proteins with the VTK heptapeptide, a surface plasmon resonance imaging (SPRi) assay was conducted. We utilized the 1:1 Langmuir binding model to analyze the interaction of DEGQ/DEGP with VTK. The results displayed that there was dose-dependent binding signals between DEGQ/DEGP and VTK, and the binding affinity represented by equilibrium dissociation rate constants (*K*_D_) of VTK toward DEGQ/DEGP proteins were 1.73 nM and 15.8 nM, respectively. Convincingly, there was no interaction signals of DEGQ/DEGP with the control HEE heptapeptide (Fig. 8e).

In addition, VTK-LL37 showed inhibitory effect on the growth of *Pseudomonas aeruginosa* (Supplementary Fig. S4b). Importantly, we found that some bacteria isolated in clinic, such as *Pseudomonas aeruginosa, Salmonella typhimurium* and *Klebsiella pneumonia*, express conserved DEGQ/DEGP proteins (Supplementary Fig. S4c,d), indicating that VTK-LL37 has potential applications in the treatment of clinical infections.

## Discussion

Clinical epidemiological investigation shows that 95% of sepsis in patients is caused by bacterial infections, and early antibacterial therapy is important for increasing the survival of patients with sepsis.^37,38^ Various bacteria, including *Salmonella, Klebsiella pneumonia*, *Listeria monocytogenes*, *Staphylococcus aureus*, *Bacillus genus*, *Hemophilus*, E. coli, *Pseudomonas aeruginosa*, *Streptococcus viridans*, and *Coagulase-negative staphylococcus*, have been reported in sepsis. However, different bacteria have different biological behaviors and sensitivities to antibiotics. As a result, the clinical manifestations, diagnostic indexes and therapeutic effectiveness of an antibiotic against sepsis are complicated. Bacterial infection is considered to be a prerequisite for the diagnosis of sepsis. In principle, individualized therapy based on accurate pathogen diagnosis could become a relatively effective strategy for sepsis treatment.

In this study, a lethal E. coli strain was isolated from the blood of mice subjected to CLP modeling and identified by DNA sequencing (Fig. 1a-e). Epidemiological studies demonstrate that gram-negative (G^-^) bacterial infection accounts for 62.2% of severe sepsis cases, among which E. coli*, Klebsiella pneumoniae* and *Pseudomonas aeruginosa* are the major pathogens causing severe sepsis in the clinic. Consistent with the epidemiology of sepsis, the lethal bacterial strain *MSI001* from CLP mice was identified to belong to the species E. coli and was closest to LR134216 and LR134231 in evolution (Fig. 1d, e).

It is well established that effective killing of pathogens in the early stage of infection is a key step for the treatment of sepsis. However, traditional antibacterial drugs are prone to become drug resistant due to their low efficiency and lack of targeting.^39,40^ Therefore, it is important to construct a new drug delivery system with a high capability of pathogen killing by improving the targeting specificity of antimicrobial drugs, mitigating multidrug resistance.^41^

Phage display technology has been widely applied in various studies, including protein-protein interactions (PPIs), antigen epitope screening,^42^ peptide screening and so on.^43^ The combination of HTS^44^ and bioinformatic technologies provides a new opportunity for phage display application in the comprehensive understanding of the biological mysteries of vast and complex biological data. The phage library contains a large number of phages expressing a great variety of exogenous peptides fused to protein III (PIII) of phages. Usually, the frequencies of the peptides displayed on phages are not even in the library. There were some reasons for the heterogenous phage amount, such as biases in library construction, variance in the use of cryptography,^45,46^ the different growth rates of phages and peptide degradation by protease.^47^

To define the bias of the phage library, we analyzed the frequency distribution of heptapeptides and found that different heptapeptides had different frequencies in the library. For example, the frequency of the heptapeptide GSAPVRS was the maximum (7,741), while the frequency of the heptapeptide DEWWLPE was the minimum (1), which confirmed the concept that the frequencies of heptapeptides presented by phages were extremely uneven. In this study, we attempted to set up a systematic approach by normalizing the heptapeptide frequency to diminish the bias in the phage library. After normalization by the heptapeptide frequencies in the phage library, we found that the frequencies of most heptapeptides had been substantially adjusted (Fig. 3; Supplementary Tables 4, 5).

To further clarify the effect of normalization on the frequency ranking, we studied the relationship between the heptapeptide frequency and the physical binding activity to lethal E. coli *MSI001*. Interestingly, we found that there was no apparent correlation between the heptapeptide frequency and the binding activity before normalization, whereas their correlation was significantly improved after normalization. Diminishing bias of the phage library could increase the opportunity to obtain high-affinity heptapeptides binding to the lethal bacterial strain.

Effective antibacterial therapy is a hopeful approach to significantly reducing the fatality of sepsis.^48^ It is crucial to develop antibacterial drugs with precise targets on pathogenic bacteria by a specific molecular design. Antimicrobial peptides (AMPs) induced by bacterial infections are small, active peptides with the capability to stimulate the innate immune activity of living organisms.^49^ Although AMPs have the characteristics of broad-spectrum antibacterial activity, overcoming multidrug resistance^50,51^ and mitigating dangerous side effects, most of these AMPs lack precise molecular targets. As a representative AMP from mammals, LL-37 shows bacteriostatic activity against various bacterial strains, such as E. coli, *Pseudomonas*, *Enterococcus*, and *Staphylococcus aureus*.

In this study, we performed phage display biopanning and acquired high-affinity heptapeptides binding to the pathogenic bacterium after normalization of the library. To improve the targeting efficiency of LL-37, we constructed a fusion peptide (VTK-LL37) composed of a high-affinity heptapeptide (VTKLGSL) and LL-37 to kill the bacteria as a “biological missile”. Interestingly, we found that the high-affinity heptapeptide and LL37 fusion peptide had stronger bacteriostatic activity than LL37 alone.

In vitro experiments demonstrated that VTK-LL37 was superior to LL37 not only in early bacteriostatic activity but also in the inhibition of biofilm formation. Furthermore, an in vivo experiment demonstrated that VTK-LL37 improved the survival of septic mice by inhibiting pathogen growth, suppressing HMGB1 production and alleviating lesions of vital organs.

Previous studies demonstrated that LL37 interacts with negatively charged groups of lipopolysaccharides (LPSs) through its positively charged group, leading to weakened toxicity to the body.^52^ LL-37 is an amphiphilic, helical AMP with a typical amphiphilic spiral structure and induces oligomer formation through its highly hydrophobic N-terminus, leading to an increased stability of LL-37 by avoiding proteolytic degradation. Both VTK-LL37 and LL37-VTK showed stronger bacteriostatic activity than other LL37 fusion peptides, such as KYY-LL37, ISS-LL37 and INS-LL37, which might be associated with their hydrophobicity and positively charges (Supplementary Table 7). The physicochemical properties of heptapeptides constitute the cornerstone for peptides targeting lethal bacterial strains.

In this study, the heptapeptide VTK was found to have binding activity to DEGP and DEGQ proteins of E. coli *MSI001*. Previous studies demonstrated that DEGP and DEGQ belong to the superfamily of heat shock proteins (HSPs). HSPs not only protect essential proteins under physiological conditions but also degrade damaged or misfolded proteins and recover raw materials for protein synthesis, thus helping cells maintain normal physiological activities.^53,54^ As HSP homologous proteases, DEGP and DEGQ were found to play a central role in protein quality control in the bacterial periplasmic space, through degrading denatured and misfolded proteins accumulated under adverse conditions.^55,56^ The increased bacterial killing effect of the VTK-LL37 fusion peptide might be explained by the cooperation between LL37 to increase membrane permeability and VTK heptapeptide with high-affinity binding to DEGP. Intriguingly, many bacteria isolated in clinic, such as *Pseudomonas aeruginosa, Salmonella typhimurium* and *Klebsiella pneumonia, have* conserved DEGQ/DEGP proteins (Supplementary Fig. S4c, d), indicating that VTK-LL37 has potential applications in the clinical control of infections. Our finding that VTK-LL37 had a potent antibacterial effect through the interaction between the VTK peptide and DEGP or DEGQ proteins might provide a novel approach with precise drug targeting for the treatment of sepsis.

In summary, we constructed a systematic approach to obtain heptapeptides targeting a bacterium by integrating phage display technology, HTS and bioinformatics. A high-affinity heptapeptide was fused to the AMP LL37 for evaluation of the antimicrobial activity of the fusion peptide VTK-LL37. We demonstrated that the antibacterial activity of VTK-LL37 was superior to LL37 both in vitro and in vivo by specifically targeting DEGP or DEGQ membrane proteins on lethal E. coli *MSI001*. Our study provides tremendous potential for the application of a systematic approach to killing pathogens by precise targeting, especially in the treatment of sepsis.

## Materials and methods

### Animals

Ten- to twelve-week-old male-specific pathogen-free (SPF) C57BL/6 mice were obtained from Southern Medical University, Guangzhou, China. All mice were housed in a temperature-controlled room with 40–70% humidity and had free access to water and food. The animal procedures were conducted in compliance with the National Institutes of Health Guide for the Care and Use of Laboratory Animals and were approved by the local Animal Care and Use Committee of Southern Medical University.

### Bacteria strain

*Pseudomonas aeruginosa* (ATCC27853) was purchased from American Type Culture Collection (ATCC) and cultured in Luria Broth (LB) medium with shaking at 37 °C. For construction of ampicillin-resistant *MSI001* strain (*MSI001-Amp*^*r*^), the pET14b plasmid carrying *Amp*^*r*^ marker gene was introduced into the wild type *MSI001* strain. The *MSI001-Amp*^*r*^ strain was cultured in LB medium supplemented with Ampicillin of 100 µg/ml at 37 °C.

### CLP model

C57BL/6 mice were randomly divided into sham and CLP groups. The CLP model was prepared with mice as described previously.^57,58^ Briefly, mice were anesthetized with 1.5% pentobarbital (0.1 ml/20 g body weight), and a midline abdominal incision 1 cm in length was made. After careful isolation, the cecum was exposed, followed by cecal ligation and puncturing twice with a 22-gauge needle. The abdominal wall was closed after the cecum was returned to the abdominal cavity. Sham-operated mice underwent the same procedure but without ligation and puncture.

### Identification of the lethal bacterial strain

Lethal bacteria were cultured in Luria Bertani (LB) broth at 37 °C. Genomic DNA was extracted by using a MiniBEST Universal Genomic DNA Extraction Kit (TaKaRa, Japan) and commercially sequenced with a PacBio Sequel platform and Illumina NovaSeq PE150 at Beijing Novogene Bioinformatics Technology Co., Ltd. The genomic DNA sequence of the lethal strain *MSI001* was blasted against the nucleotide (nt) sequence database using BLAST^+^ (v2.11.0) (https://ftp.ncbi.nlm.nih.gov/blast/executables/blast+/LATEST/) with an E value cutoff of 10^−5^. The top 500 aligning sequences and the input DNA sequence were used to identify marker genes through the Genome Taxonomy Database Toolkit (GTDB-Tk, v1.5.0, https://github.com/Ecogenomics/GtdbTk/),^59^ and the result was input into IQ-TREE (v1.5.4, http://www.iqtree.org)^60^ for phylogenetic inference. The evolution distance tree was plotted through the Interactive Tree of Life (iTOL, v6, https://itol.embl.de).^61^

### Survival study

Pathogenic bacterial clones were amplified by culturing in LB broth to an optical density at 600 nm wavelength (OD_600_) of approximately 1.0. After centrifugation at room temperature for 5 min, the bacterial precipitates were washed with Dulbecco's phosphate-buffered saline (DPBS) and resuspended in normal saline (NS) for peritoneal injection into mice. To evaluate the pathogenicity of the bacterial clones isolated from the blood or peritoneal fluid of CLP mice, the mortality of mice was observed up to 48 h after low-dose (5 × 10^6^ CFU/g body weight) or high-dose (1 × 10^7^ CFU/g body weight) bacterial injection. To study the lethal bacterial strain *MSI001*, we randomly assigned a total of 30 C57BL/6 mice evenly to 3 groups, i.e., the low-dose group, high-dose group and control group, in which the mice were subjected to peritoneal injection with 5 × 10^6^ or 1 × 10^7^ CFU/g bodyweight bacteria or an equivalent volume of NS, respectively. The survival time of mice was observed for at least 48 h after bacterial injection.

To study the therapeutic effect of synthetic peptides, C57BL/6 mice were randomly divided into 4 groups. All mice were intraperitoneally injected with bacteria (6 × 10^6^ CFU/g body weight) to reproduce a bacterial infection-induced sepsis model. After bacterial injection for 1 h, the mice were intravenously injected with the peptides LL37, VTK-LL37, or VTK at a dose of 0.2 nmol/g body weight. NS was used as control. The survival time of mice was observed for up to 168 h after bacterial injection.

### Biopanning assay

After washing with DPBS 3 times, the cultured bacteria were resuspended in 250 μL of DPBS. Ten microliters of C7C phage library (New England Bio Labs) containing 2.0 × 10^11^ PFU was added to the purified bacteria and incubated for 10 min at room temperature. Unbound phages were removed by extensive washing with Tris buffered saline with Tween 20 (TBST) containing 0.1% Tween 20, and the phages bound to bacteria were eluted by glycine-HCl solution. Phage titration was performed by fold dilution, and phage DNA was extracted using a Quick Gene DNA Tissue Kit for M13 (BioTeke, China) following the procedure supplied by the manufacturer.

### Construction of His-tagged fusion protein expression plasmids

After enzyme digestion with BamHI, the pET14b/His-EGFP plasmid was linearized and used as the template for PCR amplification.^62^ The DNA sequences encoding heptapeptides were fused to the His-EGFP-encoding sequence to construct the expression plasmid pET14b/His-EGFP-C7C by PCR using the common forward primer 5’-TGCTAAGGATCCCAAAGCCCGAAAGGAAGCTGAGTTGG-3’ and a corresponding reverse primer (Supplementary Table 6). The PCR was conducted with initial denaturation at 94 °C for 5 min, followed by 30 cycles of denaturation at 98 °C for 30 s, annealing at 62 °C for 30 s and extension at 68 °C for 1 min, with a final extension at 68 °C for 10 min. The amplified products were resolved by electrophoresis on a 1% agarose gel. PCR products were phosphorylated, ligated, and then transformed into E. coli BL21(DE_3_) for the production of His-EGFP-C7C proteins.

### Affinity validation of binding peptides

The E. coli *MSI001* bacterium was pelleted by centrifugation and washed with DPBS 3 times. Then, the His-EGFP and His-EGFP-C7C fusion proteins were added to 600 µL of a suspension containing 3 × 10^10^ CFU bacteria with a final concentration of 1 µmol/L. After incubation for 4 h, the sample was centrifuged at 4 °C and 10,000 rpm for 3 min, the supernatant was discarded, and the sediment was washed with DPBS 3 times and resuspended in 200 µL of DPBS. Finally, the fluorescence intensity of the protein binding to the bacteria was measured using a Spectramax M_5_ spectrometer (Molecular Devices, San Jose, CA, USA). Theoretically, a heptapeptide with a relatively high frequency should have a relatively large fluorescence intensity value. To test the correlation between the frequency of the heptapeptide and the fluorescence intensity, we performed bivariate correlation analysis with SPSS (v20.0).

### In vitro antibacterial assay

The synthetic peptides LL37, VTK-LL37, LL37-VTK, KYY-LL37, LL37-KYY, ISS-LL37 and INS-LL37 were added to a bacterial suspension (1 × 10^3^ CFU) at final concentrations of 0.25 μmol/L, 0.5 μmol/L, and 1 μmol/L. Sterile DPBS was used as control. The bacteria were incubated at 37 °C for 8 h, and the culture suspension was sequentially obtained for serial dilution with LB. The diluted suspension was plated on agar plates for culturing at 37 °C overnight, followed by calculating the bacterial colony-forming units (CFU).

### Biofilm detection

When the OD_600_ of the bacterial suspension reached 1.0, the bacteria were transferred to a 96-well plate, and the VTK-LL37 or LL37 peptide was added at final concentrations of 0.5 μmol/L, 1 μmol/L, 2 μmol/L, or 4 μmol/L, followed by incubation at 37 °C. Replacement with fresh LB broth, as well as the peptides VTK-LL37 and LL37, was performed every 24 h. After culturing for 3 days, the suspended bacteria were discarded, and the plate was washed with DPBS 3 times. Bacterial biofilms were observed by microscopy and quantitated by spectrometry. In detail, after air-drying for 10 min, the biofilm attached to the bottom of a 96-well plate was stained with 1% crystal violet for 20 min. Then, the plate was washed with sterile water 3 times, followed by air drying. The precipitates in the plate were dissolved in 95% ethanol, followed by detection of the absorbance at 570 nm (A570) with a Spectramax M_5_ spectrometer.

### In vivo antibacterial assay

Bacteria in the logarithmic growth stage were collected and washed with DPBS 3 times. Thirty-two C57BL/6 mice were evenly divided into 4 groups, i.e., the sepsis group, VTK-LL37 group, LL37 group and control group. Mice in the sepsis, LL37 and VTK-LL37 groups were intraperitoneally injected with E. coli *MSI001* at a dose of 6 × 10^6^ CFU/g bodyweight to reproduce the sepsis model. The control mice received an injection of an equivalent volume of NS. One hour after sepsis modeling, the mice of VTK-LL37 and LL37 groups were subjected to tail vein injection (TVI) of VTK-LL37 or LL37 at a dose of 0.2 nmol/g body weight, and the other group mice were injected with an equivalent volume of NS. Twelve hours later, blood was sequentially obtained from mice and diluted with LB broth. The diluted samples were plated on agar plates and cultured at 37 °C overnight to count the CFUs.

### Histopathological examination

Twelve hours after modeling, the mice were anesthetized with 1.5% pentobarbital (0.1 ml/20 g), and the liver, kidney, heart and lung tissues were collected for routine pathological examination. The tissue samples were fixed with 4% formalin for 12 h and embedded in paraffin, followed by 3-μm-thick sectioning. Hematoxylin and eosin (H&E) staining was performed for observation under an Axio Imager Z2 microscope (Zeiss, Germany).^63^

### Histopathological evaluation

Tissue sections of vital organs were stained with H&E and observed by microscopy. Multiple organ injury representations, including interalveolar septum thickened in the lung, tubular epithelial cell swelling in the kidney, inflammatory cell infiltration in the liver and heart. The detail criteria were based on a previously established method.^64^

### Pull-down assay for heptapeptides

Membrane proteins were obtained from E. coli *MSI001* by using a bacterial membrane protein extraction kit (BesBio, Shanghai, China). Biotinylated VTK or HEE heptapeptide was incubated with streptavidin-coated magnetic beads (Millipore, LSKMAGT) for 1 h. The unbound biotinylated heptapeptides were removed by washing with PBS 3 times. The heptapeptide-bound magnetic beads were incubated with the bacterial membrane proteins at 37 °C for 4 h to pull down the membrane proteins interacting with the heptapeptide.

### Sample preparation for LC-MS/MS

Protein samples were prepared in accordance with a standard protocol for filter-aided sample preparation (FASP). Briefly, the protein sample (100 µg) in a Vivacon 500 filtrate tube (Sartorius Stedim Biotech GmbH, Goettingen, Germany) was mixed with 300 µL of 8 mol/L urea in 0.1 mol/L NH_4_HCO_3_ (pH 8.5), followed by centrifugation at 14,000 × *g* at room temperature for 15 min. The sample was washed with 300 µL of 8 mol/L urea in 0.1 mol/L NH_4_HCO_3_ (pH 8.5), and irrelevant components were removed by centrifugation. After repeating this procedure three times, the sample was washed with 300 µL of 0.1 mol/L NH_4_HCO_3_ (pH 8.5).

The sample was incubated with 5 μL of 0.5 mol/L dithiothreitol (DTT) in 250 μL of 100 mmol/L NH_4_HCO_3_ solution at 56 °C for 30 min, followed by incubation with 10 μL of 0.5 mol/L iodoacetamide (IAA) in the dark for 30 min. After three washes with 300 μL of 100 mmol/L NH_4_HCO_3_, the protein sample was digested in a filter with 2 μg of trypsin (Cat# v5280, Promega, Madison, WI, USA) in 300 μL of 0.1 mol/L NH_4_HCO_3_. After incubation at 37 °C for 16–18 h, the peptides were collected through centrifugation at 14,000 × *g* for 15 min. Finally, the concentrated peptides were desalted through a C18 column (Cat# S181001; Agela Technologies, Torrance, CA, USA).

### LC-MS/MS analyses

The peptide sample (1 µg) was analyzed by using an EASY-nLC1200 instrument connected to an Orbitrap fusion mass spectrometer (Thermo Scientific, Waltham, MA, USA). The peptides were separated by a linear gradient of 5–30% acetonitrile (ACN) with 0.1% formic acid (FA) at 300 nL/min for 48 min, which was then linearly increased to 100% ACN over 7 min and maintained at 100% for 5 min. The column was re-equilibrated with 6 µL of 0.1% FA. The data-dependent acquisition (DDA) scheme was performed with a full MS survey scan in the range of m/z 350 to 1500 at a resolution of 120,000 full width at half maximum (FWHM) and the automatic gain control (AGC) set to 2.0E5, followed by a top speed data acquisition model at a resolution of 30,000 FWHM with the AGC set to 5.0E4.

MS raw data were processed by MaxQuant software (v1.6.10.43) (https://www.maxquant.org/maxquant/, Max Plank Institute of Biochemistry, Planegg, Germany), and fragments were searched against the UniProt database of *Mus musculus*. The false discovery rate (FDR) was set to 0.01, and a minimum length of 7 AAs for peptides was specified. The search results were processed with Perseus software (v1.6.10.50) (https://www.maxquant.org/perseus/).

### Bioinformatic analysis for heptapeptide binding proteins

CPCoA based on the Bray-Curtis distance matrix was conducted by online ImageGP software (http://www.ehbio.com/ImageGP/). Visual hierarchical cluster analysis was performed after generating volcano plots and heatmaps in ImageGP software (http://www.ehbio.com/ImageGP/index.php/Home/Index/index.html). Protein sequence alignment was performed on UniProt (https://www.uniprot.org/).

### SPRi analysis

To measure the binding affinity of peptides with targeted proteins, a PlexArray HT A100 (Plexera, USA) Surface Plasmon Resonance imaging (SPRi) system was used to monitor the whole procedure in real time. Briefly, a chip with a well-prepared peptide microarray was assembled with a plastic flow cell for sample loading. The operation details for the PlexArray HT were as described previously.^65^ The purified protein samples were prepared at determined concentrations in PBS running buffer, while glycine-HCl buffer (10 mmol/L, pH 2.0) was used for regeneration. Binding data were collected and analyzed by SPR Data Analysis Module software (Plexera, USA). The *K*_D_ values were obtained using a 1:1 Langmuir binding model via the BIAevaluation Software ver4.1 (Biacore AB, Germany).

### Statistical analysis

Results are expressed as the mean ± SEM except other description. For statistical analysis, significant differences between groups which pass both normality (Shapiro-Wilk test) and equal variance test (Levene’s test) were evaluated using the one-way ANOVA follow by Bonferroni post hoc test for multiple comparisons, or the unpaired two-tailed Student’s *t*-test for comparison between two groups. Welch correction was used if the data failed the test of equal variance and followed by Dunnett T3 post hoc test for multiple comparisons. Survival differences were analyzed by Kaplan-Meier survival curves. Bivariate regression analysis was performed to determine the correlation between two variables. All statistics were analyzed using Statistical Package for the Social Sciences (SPSS) software (v22.0), and *P* < 0.05 was considered statistically significant.

## Supplementary information

SUPPLEMENTAL MATERIAL (marked-up)

SUPPLEMENTAL MATERIALS

## Data Availability

The data used in the study are available from the corresponding authors upon reasonable request.
