# Supplementary material for: Identification of heptapeptides targeting a lethal bacterial strain in septic mice through an integrative approach
Source: Signal Transduct Target Ther. 2022 Jul 25;7:245. doi: 10.1038/s41392-022-01035-6 (PMC9309159; doi:10.1038/s41392-022-01035-6)
Supplement: Supplementary file 1 — SUPPLEMENTAL MATERIAL (marked-up) [file 41392_2022_1035_MOESM1_ESM.doc]

Supplementary Materials for

Identification of heptapeptides targeting a lethal bacterial strain in septic mice through an integrative approach

Xiaoyan Zhang^1#^, Shan Li^1#^, Haihua Luo^1#^, Shuyue He^1^, Huangda Yang^1^, Lei Li^1^, Tian Tian^1^, Qizheng Han^1^, Jiacong Ye^1^, Chenyang Huang^1^, Aihua Liu^2^*, Yong Jiang^1^*

Correspondence to: jiang48231@163.com

**This PDF file includes:**

Materials and Methods

Figures S1 to S8

Tables S1 to S7

**Materials and Methods**

**HTS**

Polymerase chain reaction (PCR) was performed to amplify the phage DNA fragments containing the sequences encoding random heptapeptides with a pair of specific primers (Forward: 5'-CACCTCGAAAGCAAGCTGAT-3'; Reverse: 5'-CAACGCCTGTAGCATTCCAC-3') (Fig. 2b). The PCR was set as initial denaturation at 95°C for 5 min, followed by 25 cycles of denaturation (95°C, 30 s), annealing (52°C, 30 s) and extension (68°C, 30 s), with a final extension at 68°C for 5 min. The amplified products were resolved by electrophoresis on a 2% agarose gel. The recovered DNA fragments were commercially sequenced by using a MiSeq instrument (Illumina) with a 250-bp paired-end-read format at Novogene Bioinformatics Technology Co., Ltd. (Beijing, China).

**Analysis of the AA composition and physicochemical properties of heptapeptides**

The AA composition percentages in full-length heptapeptides and at each site of the heptapeptides were calculated. According to the physical properties, all the AAs were classified as hydrophobic and hydrophilic and were further divided into acidic, neutral and alkaline groups. Aspartic acid (D) and glutamic acid (E) are acidic AAs; serine (S), threonine (T), asparagine (N), glycine (G), glutamine (Q), tyrosine (Y) and cysteine (C) are neutral AAs; and arginine (R), histidine (H) and lysine (K) are alkaline AAs. The hydrophilic AAs were proline (P), leucine (L), alanine (A), methionine (M), valine (V), isoleucine (I), phenylalanine (F) and tryptophan (W). The net charge of the binding peptides was calculated with online open-source software (http://protcalc.sourceforge.netc). The PI and GRAVY of the binding peptides were determined online at http://www.endmemo.com/bio/proie.php and http://www.gravy-calculator.de/index.php, respectively. All the data were collected and statistically analyzed to characterize the profiles of the physicochemical properties of the binding peptides.

**Peptide synthesis**

The peptides LL37, VTK-LL37, LL37-VTK, KYY-LL37, LL37-KYY, ISS-LL37, INS-LL37 and biotin-labeled VTK and HEE heptapeptides were commercially synthesized by Shanghai Botai Company (Shanghai, China). The AA sequences of the peptides are shown in Supplementary Table 7. Chromatograms and mass spectra of these peptides are shown in Supplementary Figure 3.

**HMGB1 quantitation**

HMGB1 in the serum of mice was quantitated by using an ELISA kit (Cat# CSB-E08225m) purchased from CUSABIO (Wuhan, China) according to the manufacturer’s instructions.

**Detection of ALT, AST, LDH and CRE levels in serum**

The activities of alanine aminotransferase (ALT), aspartate aminotransferase (AST) and lactate dehydrogenase (LDH), and Creatinine (Cre) in serum were determined by using the commercially available kits from Jiancheng Institute of Bioengineering, Inc. (Nanjing, China) following the manufacturer’s instructions.


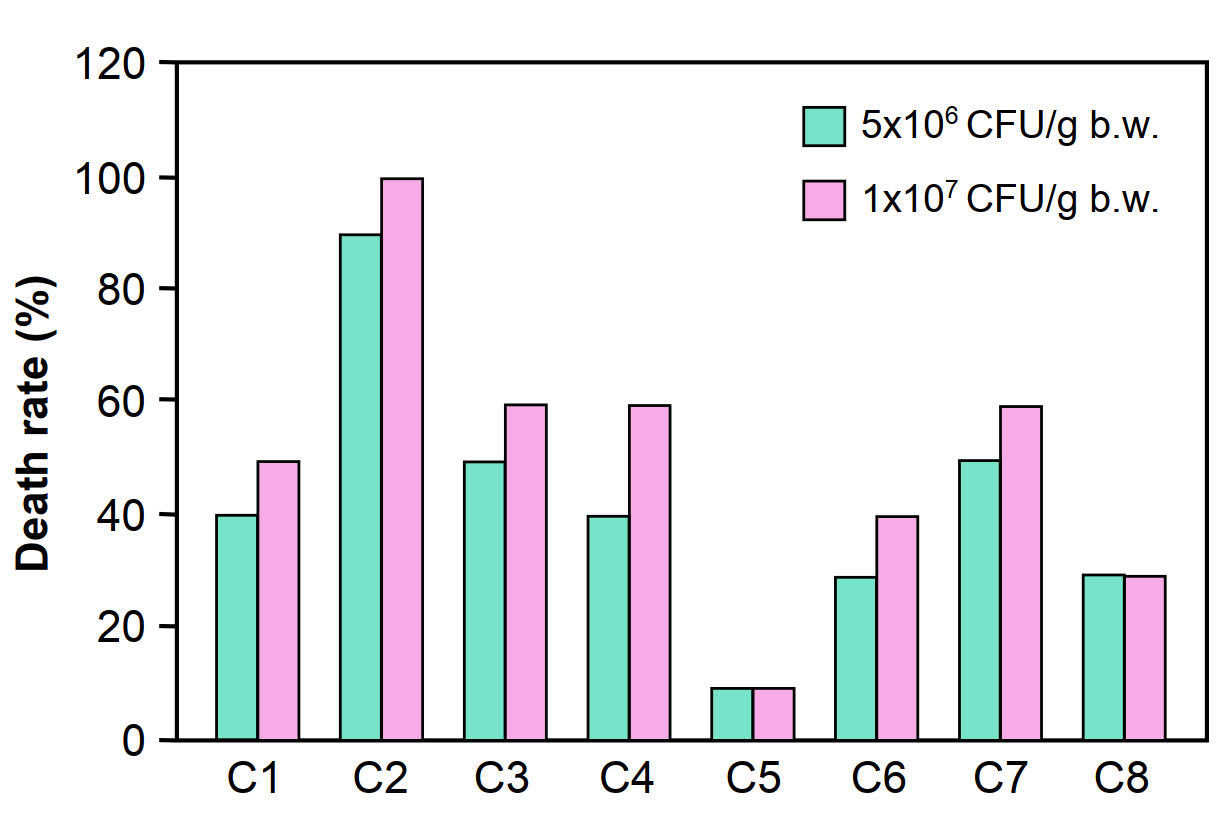


**Figure S1. The death rate of mice subjected to bacterial strains isolated from CLP mice.** The bacterial colonies were cultured and isolated from blood (C1-C4) or peritoneal fluid (C5-C8) of CLP mice. WT C57 mice were peritoneally injected with bacteria of 5×10^6^ CFU/g body weight or 1×10^7^ CFU/g body weight. Death rate of mice were observed for 48 h after bacterial injection. *n*=10.


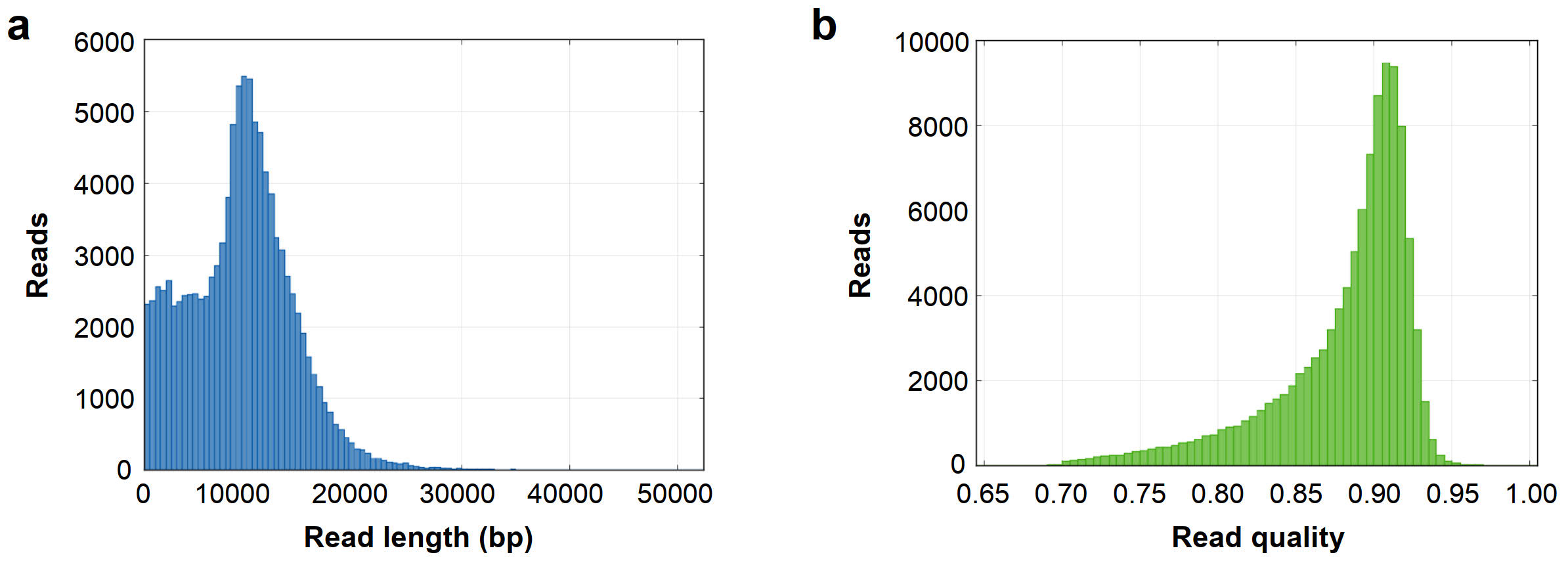


**Figure S2. Basic information of DNA sequencing of the *E. coli MSI001* strain. a** Read length distribution of the DNA sequencing of the *MSI001* bacterial strain. **b** Read quality of the DNA sequencing of the *MSI001* bacterial strain.

**
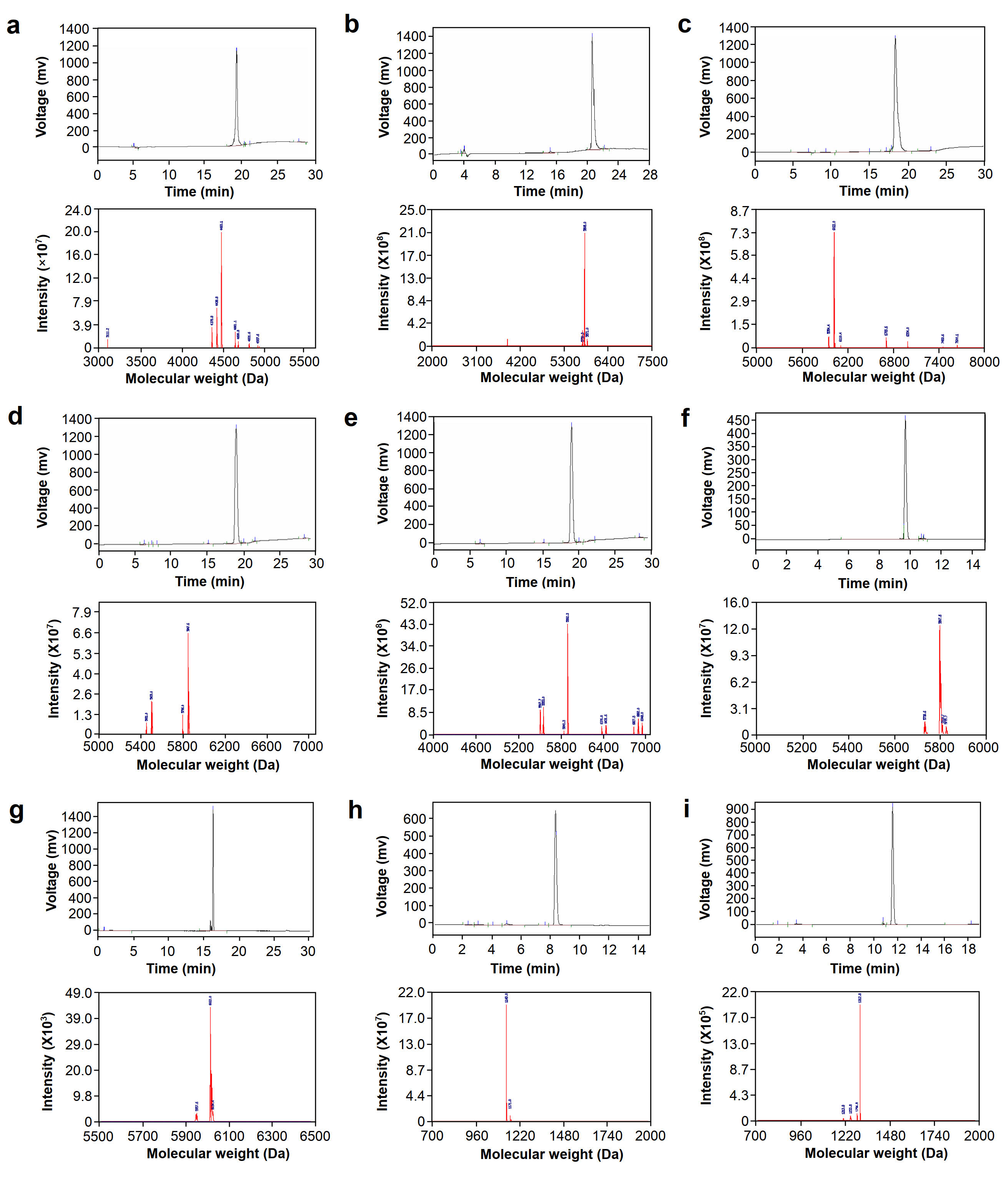
**

**Figure S3. Chromatograms and peptide mass spectrometry of the synthesized peptides. a-i** Chromatogram (upper panel) and mass spectrum (lower panel) of synthesized peptides LL-37 (**a**), VTK-LL37 (**b**), KYY-LL37 (**c**), ISS-LL37 (**d**), INS-LL37 (**e**), LL37-VTK (**f**), LL37-KYY (**g**), biotin-CVTKLGSLC (**h**), biotin-CHEEWLPAC (**i**).

**
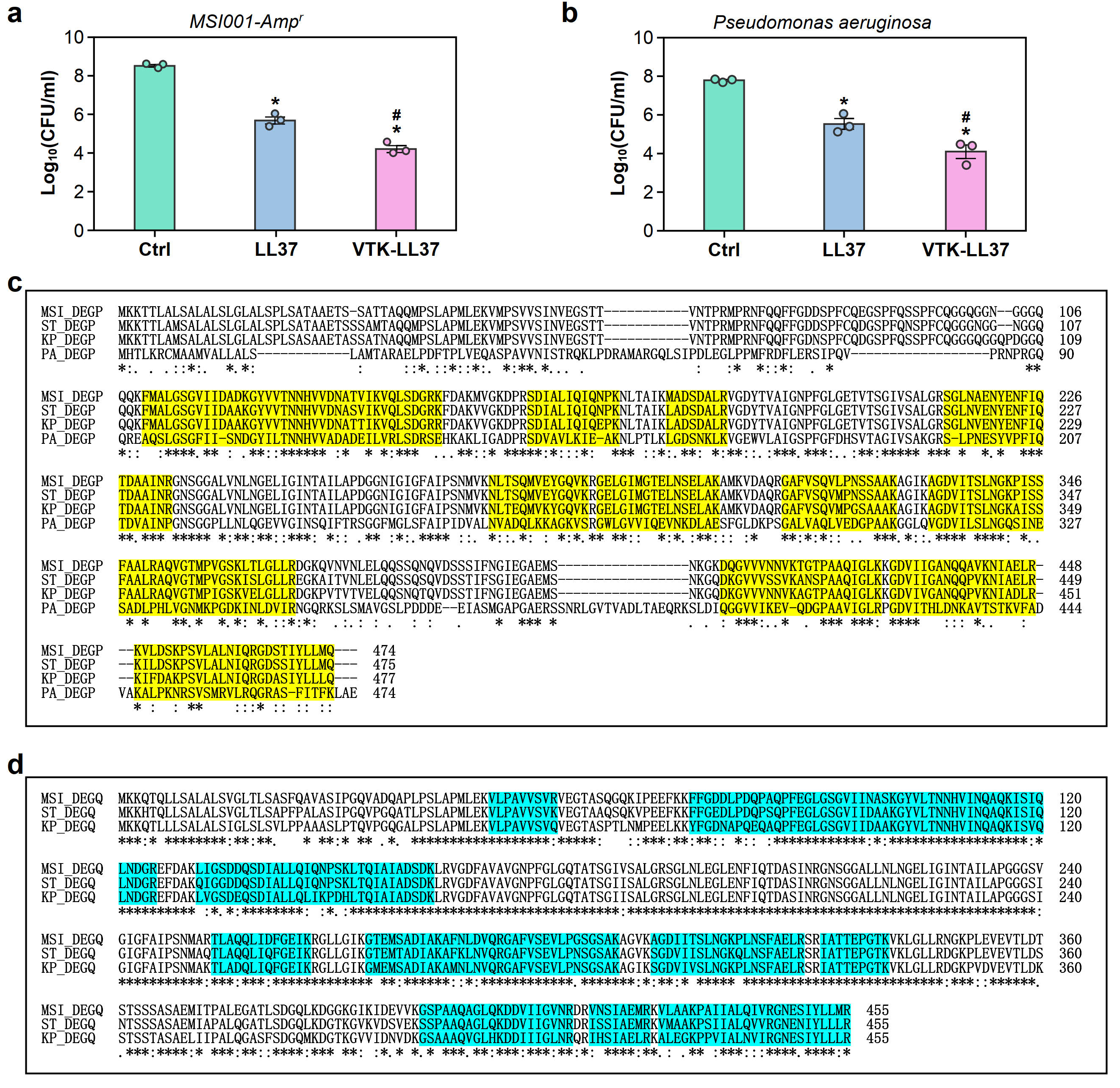
**

**Figure S4. Inhibitory effect of LL37 and VTK-LL37 fusion peptides on bacterial growth.** **a** Effect of VTK-LL37 on the growth of Ampicillin resistant (*Amp^r^*) *MSI001* bacteria. The bacterial strain *MSI001* was transfected with pET14b plasmid to produce an Ampicillin resistant (*Amp*^r^) *MSI001* strain. The VTK-LL37 or LL37 peptide was added to the cultured *MSI001-Amp^r^* bacteria (1×10^3^ CFU/ml) with a final concentration of 1 μmol/L. After incubation with the VTK-LL37 or LL37 peptides at 37°C for 3 hours, bacterial colonies were counted. **b** Effect of VTK-LL37 on the growth of *Pseudomonas aeruginosa.* The bacterial colonies of *Pseudomonas aeruginosa* were counted as above. Sterile DPBS was used as control. *n*=3. **P*<0.05, compared to control group; ^#^*P*<0.05, compared to LL37 group. **c, d** Protein sequence alignment of DEGP (**c**) and DEGQ (**d**) of different bacterial strains including *E.coli* *MSI001* (MSI), *Pseudomonas aeruginosa* (PA, GenBank Accession^#^ QKS39086), *Salmonella typhimurium* (ST, GenBank Accession^#^ AAL19173) and *Klebsiella pneumonia* (KP, GenBank Accession^#^ ABR75636). The genomic DNA of *E.coli* *MSI001, Salmonella typhimurium* and *Klebsiella pneumonia* encode both DEGP and DEGQ, while *Pseudomonas aeruginosa* only expresses DEGP. The AAs with background color represent peptide fragments identified by mass spectrometry.

**
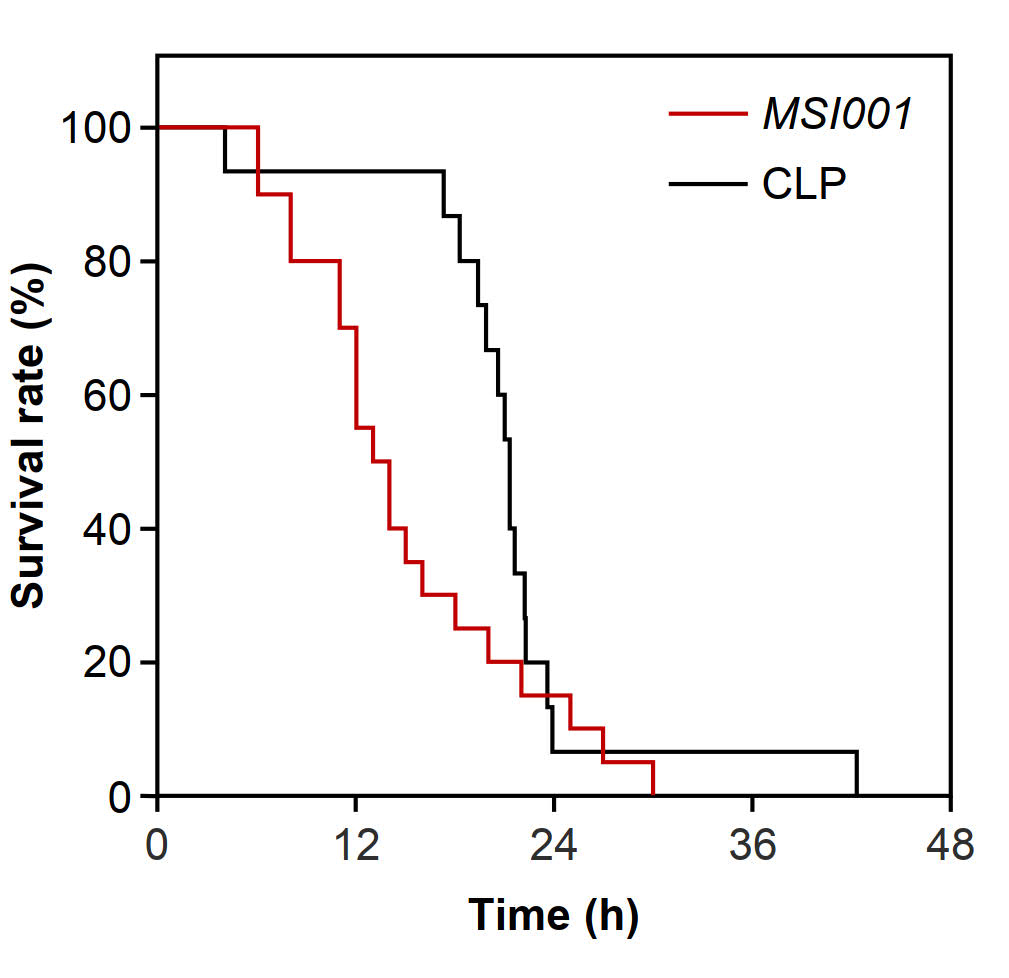
**

**Figure S5. Comparison on the survival rate of septic mice subjected to intraperitoneal injection of *MSI001* bacteria (6×10^6^ CFU/g body weight) or CLP modeling**. The mice were observed for survival up to 48 h after modeling. *n*=20, for *MSI001* group; *n*=15, for CLP group.


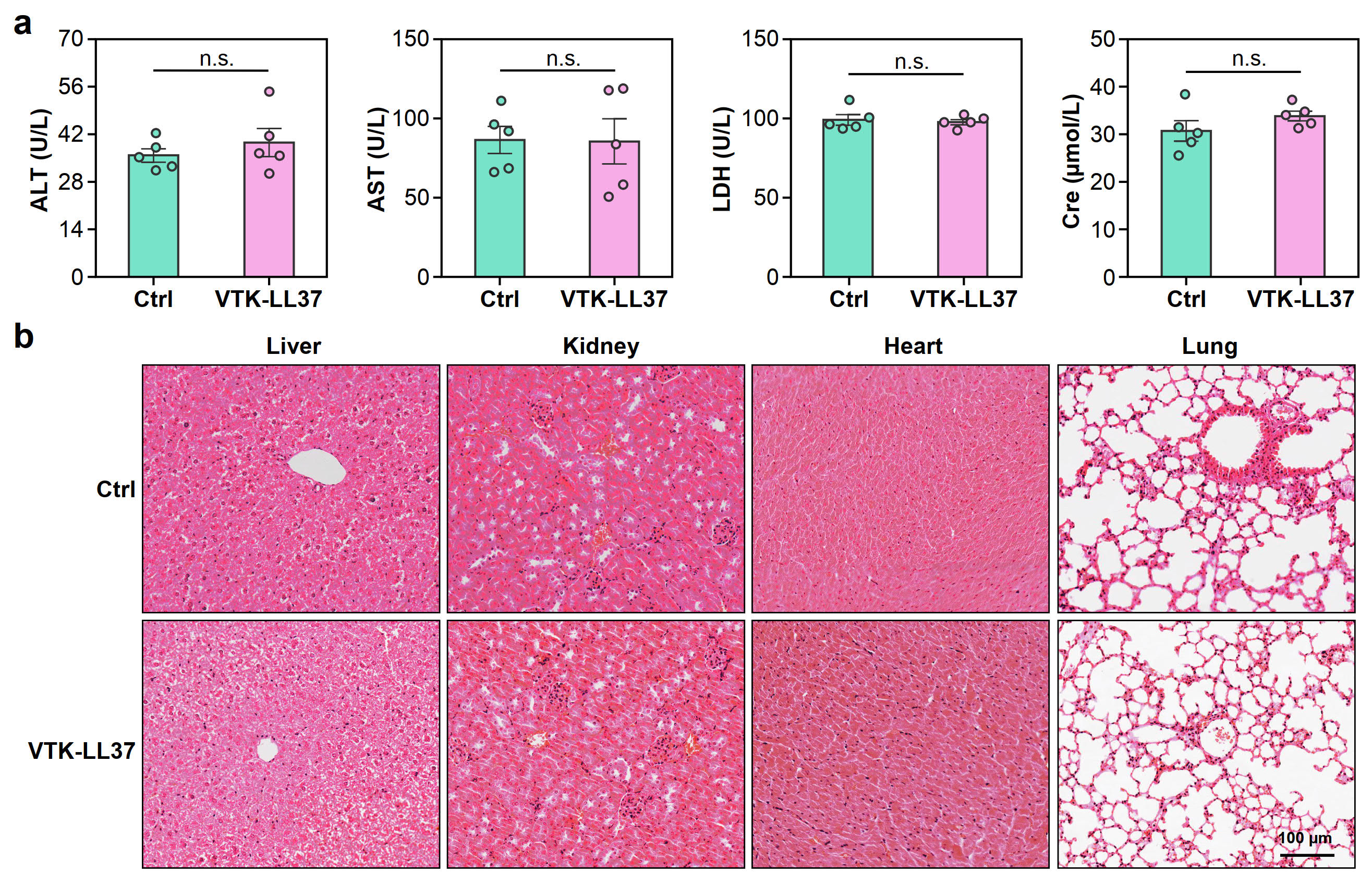


**Figure S6. Therapeutic dosage of VTK-LL37 without perceivable side effects on mice. a** The effects of VTK-LL37 on the function of vital organs. After intravenous injection with VTK-LL37 peptide of 10× therapeutic dosage (2 nmol/g body weight) for 12 h, the blood of mice were collected for detection of ALT, AST, LDH and creatinine in the serum. **b** Histopathological evaluation of VTK-LL37 on the tissue structure of vital organs. The liver, kidney, heart and lung tissues were collected for routine pathological examination at 12 h after intravenous injection with VTK-LL37 peptide at a dosage of 2 nmol/g body weight. Hematoxylin and eosin (HE) staining was performed for histopathological evaluation by microscopy. *n*=5; n.s.=not significant.


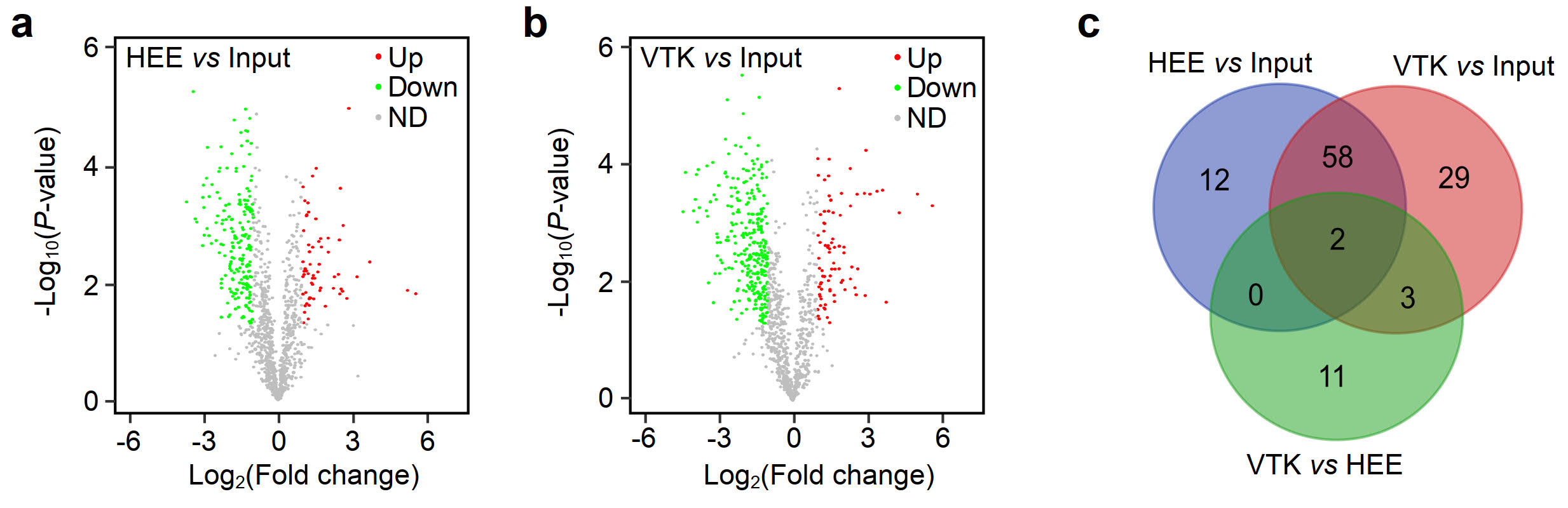


**Figure S7. The differential proteins of VTK group and HEE group in comparison with input. a** Volcano plot of proteins from HEE group and input. **b** Volcano plot of proteins from VTK group and input. Red and green dots represent up- and down-regulated proteins with |fold change (FC)|>1.5 and *P*-value<0.05 compared to the control, respectively. The horizontal axis represents the log_2_(FC) of bacterial proteins pulled-down by HEE or VTK heptapeptide in comparison with input. **c** Venn diagram of differential proteins between the HEE, VTK and input groups with |FC|>1.5 and *P*-value<0.05.


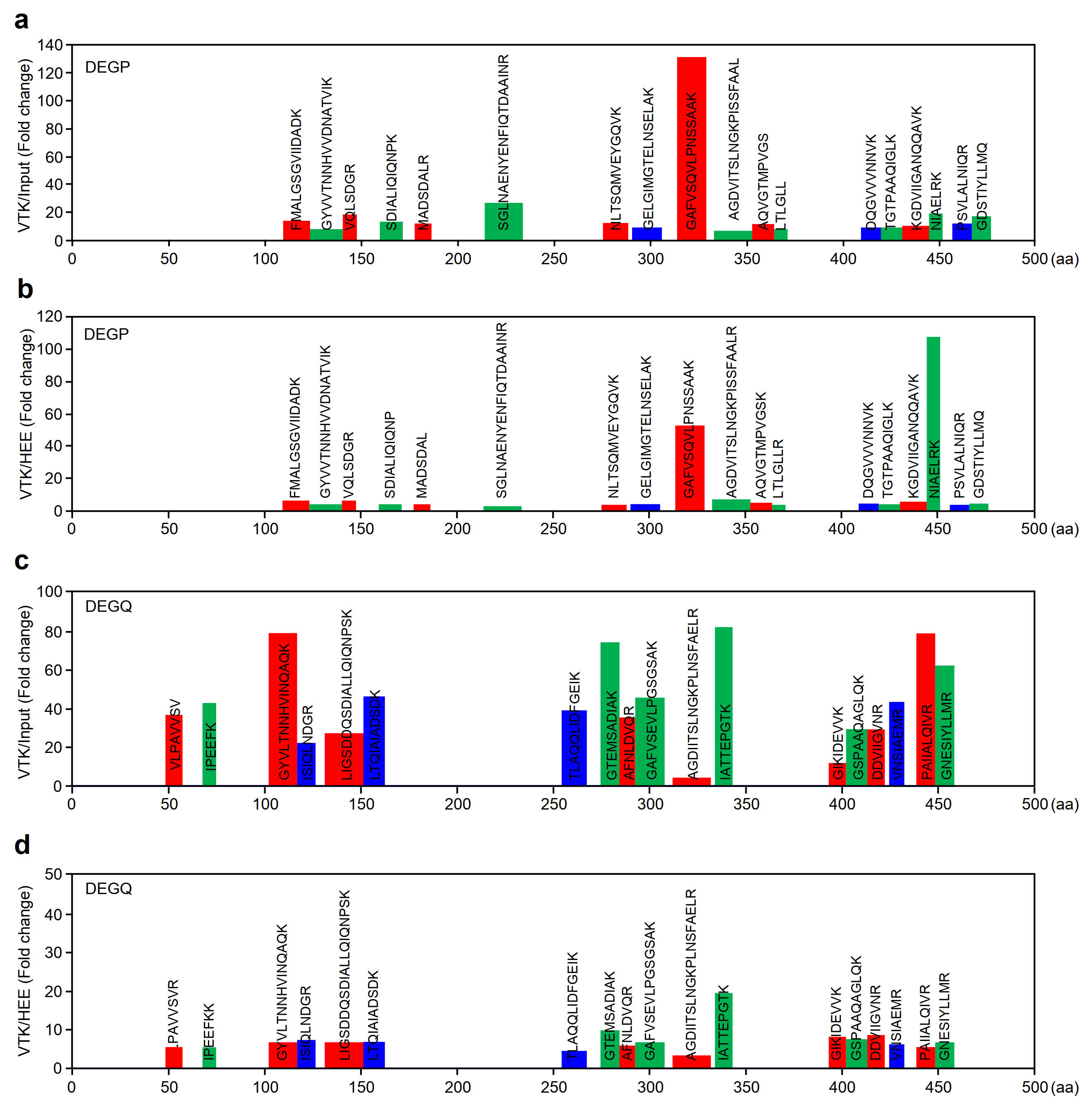


**Figure S8. Enrichment of peptide fragments of DEGP and DEGQ identified by mass spectrometry. a** Relative abundance of peptide fragments of DEGP identified by mass spectrometry of the VTK group in comparison with input. **b** Relative abundance of peptide fragments from DEGP identified by mass spectrometry of the VTK group in comparison with the HEE group. **c** Relative abundance of peptide fragments from DEGQ of the VTK group in comparison with input. **d** Relative abundance of peptide fragments from DEGQ of the VTK group in comparison with the HEE group. The width of each bar represents corresponding peptide length (AA) and the height represents enrichment (fold change) of peptide of DEGP or DEGQ in comparison with input or the HEE group. The AA sequence of each peptide is shown on the corresponding bar.

**Table S1. Summary information for phage DNA sequencing data.**

| **Samples** | **Raw Reads** | **Clean Reads** | **Raw base (G)** | **Clean base (G)** | **GC Content (%)** |
| --- | --- | --- | --- | --- | --- |
| Library phages | 93,253,550 | 89,409,753 | 46.63 | 44.71 | 39.97 |
| Binding phages | 8,896,080 | 8,805,456 | 4.45 | 4.40 | 39.45 |

**Table S2. Frequency distribution of displaying heptapeptides from the phage library.**

| **No.** | **Heptapeptide** | **Frequency** |  | **No.** | **Heptapeptide** | **Frequency** |
| --- | --- | --- | --- | --- | --- | --- |
| 1 | GSAPVRS | 7741 |  | 16 | VPSKPGL | 2638 |
| 2 | LTAKHMQ | 7257 |  | 17 | GLTSPLA | 2369 |
| 3 | FATRADH | 4656 |  | 18 | TQQTTSL | 2359 |
| 4 | IHSPTAL | 3484 |  | 19 | QHGVELS | 2255 |
| 5 | IAMWASN | 3370 |  | 20 | MEENSRY | 2236 |
| 6 | NSHTQGK | 3323 |  | 21 | NWMINKE | 2226 |
| 7 | PKGDENT | 3090 |  | 22 | RADLLEM | 2185 |
| 8 | NADQTNI | 3080 |  | 23 | SAQQPAS | 2181 |
| 9 | NMHTPMV | 3023 |  | 24 | THMPVLS | 2175 |
| 10 | EWAGPWT | 2956 |  | 25 | LNRYVAD | 2159 |
| 11 | NDITQNH | 2925 |  | 36 | LAYAHHT | 2137 |
| 12 | YARPGES | 2899 |  | 27 | ARVHSLG | 2091 |
| 13 | NIKSSHV | 2840 |  | 28 | QWRHTLE | 2080 |
| 14 | IDHRGAA | 2769 |  | 29 | GYGEPDK | 2043 |
| 15 | VNQSTPR | 2693 |  | 30 | ERASGLY | 2029 |

**Table S3. Frequency distribution of binding heptapeptides.**

| **No.** | **Heptapeptide** | **Frequency** |  | **No.** | **Heptapeptide** | **Frequency** |
| --- | --- | --- | --- | --- | --- | --- |
| 1 | IHSPTAL | 5124 |  | 15 | GPTAKYI | 2867 |
| 2 | NAGHLSQ | 4414 |  | 16 | PSTVPWS | 2856 |
| 3 | NIKSSHV | 4296 |  | 17 | VPSKPGL | 2826 |
| 4 | NMHTPMV | 4232 |  | 18 | NSHTQGK | 2522 |
| 5 | ISSSINH | 3804 |  | 19 | SVGYDRN | 2487 |
| 6 | TLKNLAL | 3787 |  | 20 | SENSPLL | 2478 |
| 7 | LTAKHMQ | 3715 |  | 21 | SKEATPF | 2445 |
| 8 | LAYAHHT | 3457 |  | 22 | LDHSSKL | 2182 |
| 9 | NWMINKE | 3267 |  | 23 | NTSTMLH | 2181 |
| 10 | FATRADH | 3089 |  | 24 | EQAHKLH | 2172 |
| 11 | DGLAKNS | 3028 |  | 25 | TTKLPNS | 2066 |
| 12 | PKGDENT | 2982 |  | 26 | NANPWRL | 2054 |
| 13 | SAQQPAS | 2966 |  | 27 | LNRYVAD | 2027 |
| 14 | NDTKQGN | 2904 |  |  |  |  |

**Table S4. Frequency ranking of binding heptapeptides without normalization.**

| **Un-R** | **N-R** | **Heptapeptide** |  | **Un-R** | **N-R** | **Heptapeptide** |
| --- | --- | --- | --- | --- | --- | --- |
| 1 | 564599 | IHSPTAL |  | 51 | 583642 | TMNSPSK |
| 2 | 306338 | NAGHLSQ |  | 52 | 658449 | RADLLEM |
| 3 | 554674 | NIKSSHV |  | 53 | 228846 | SAYDRPL |
| 4 | 567321 | NMHTPMV |  | 54 | 568114 | PLSSGYA |
| 5 | 313845 | ISSSINH |  | 55 | 634990 | LSAETNR |
| 6 | 284138 | TLKNLAL |  | 56 | 329464 | MDNHLAV |
| 7 | 667530 | LTAKHMQ |  | 57 | 567996 | HDTQPRT |
| 8 | 552154 | LAYAHHT |  | 58 | 313876 | HYNTPYA |
| 9 | 564619 | NWMINKE |  | 59 | 648793 | ADPSSQT |
| 10 | 658354 | FATRADH |  | 60 | 646982 | QKSINLI |
| 11 | 328251 | DGLAKNS |  | 61 | 309745 | TYLNSAK |
| 12 | 633564 | PKGDENT |  | 62 | 209946 | GLHNMAV |
| 13 | 568005 | SAQQPAS |  | 63 | 636153 | NWHKSLP |
| 14 | 564669 | NDTKQGN |  | 64 | 666314 | GLTSPLA |
| 15 | 564620 | GPTAKYI |  | 65 | 329454 | HGNLNAF |
| 16 | 541373 | PSTVPWS |  | 66 | 665504 | QHGVELS |
| 17 | 583208 | VPSKPGL |  | 67 | 313875 | NKFSLAS |
| 18 | 642780 | NSHTQGK |  | 68 | 583325 | YGLLRTG |
| 19 | 306190 | SVGYDRN |  | 69 | 634262 | GSSTSTT |
| 20 | 307305 | SENSPLL |  | 70 | 313815 | SNFDTRS |
| 21 | 574274 | SKEATPF |  | 71 | 634273 | MHLGPFR |
| 22 | 284139 | LDHSSKL |  | 72 | 541579 | PQLTNMS |
| 23 | 577274 | NTSTMLH |  | 73 | 288109 | ENKHTWG |
| 24 | 546845 | EQAHKLH |  | 74 | 84 | TTLRVPL |
| 25 | 568088 | TTKLPNS |  | 75 | 583274 | LPTKFRS |
| 26 | 577594 | NANPWRL |  | 76 | 583278 | DRMPRGA |
| 27 | 633858 | LNRYVAD |  | 77 | 658375 | PVTPGSP |
| 28 | 577120 | VDTRIQM |  | 78 | 543483 | PTSQRDN |
| 29 | 577600 | MNQASMS |  | 79 | 547710 | KSTELSM |
| 30 | 660304 | NADQTNI |  | 80 | 324887 | QTNDAQM |
| 31 | 546812 | NNSGKQL |  | 81 | 278969 | QSFFTVS |
| 32 | 660332 | NDITQNH |  | 82 | 329378 | VNQGSIG |
| 33 | 636256 | GYGEPDK |  | 83 | 329364 | GTLHWNK |
| 34 | 582709 | LKPHSAD |  | 84 | 633545 | LSTTEGY |
| 35 | 541256 | LSSTTKN |  | 85 | 666725 | FPTGTYW |
| 36 | 541285 | SGDSKPS |  | 86 | 583591 | EGSSNHD |
| 37 | 313653 | NATLYKD |  | 87 | 641719 | SSKHEAT |
| 38 | 577671 | KHYLHEG |  | 88 | 647574 | GTSLYAT |
| 39 | 581465 | SLFSKNY |  | 89 | 567823 | VDHERQN |
| 40 | 583505 | NMPKLHT |  | 90 | 642774 | GGSMLSS |
| 41 | 554629 | EKGPGIS |  | 91 | 553619 | PGMHAQT |
| 42 | 666742 | YARPGES |  | 92 | 658486 | MSLNAVG |
| 43 | 583479 | ETGRWMH |  | 93 | 286113 | NTDHNAR |
| 44 | 583590 | LSGVTEG |  | 94 | 541409 | IWHPNNA |
| 45 | 633565 | HLLETHT |  | 95 | 327612 | NTHREPQ |
| 46 | 646955 | ERASGLY |  | 96 | 583572 | IAMWASN |
| 47 | 636288 | IPLITHY |  | 97 | 722563 | GMAHGGK |
| 48 | 634421 | ESRANWL |  | 98 | 573521 | NSISLAL |
| 49 | 568001 | DAHYEKS |  | 99 | 283153 | TTSDAHR |
| 50 | 308091 | KDFSTRH |  | 100 | 663720 | TTWALAH |

Un-R: unnormalized rank; N-R: normalized rank.

**Table S5. Frequency ranking of binding heptapeptides with normalization.**

| **N-R** | **Un-R** | **Heptapeptide** |  | **N-R** | **Un-R** | **Heptapeptide** |
| --- | --- | --- | --- | --- | --- | --- |
| 1 | 106 | QTYHSGH |  | 51 | 1959 | KDGTSKI |
| 2 | 298 | VTKLGSL |  | 52 | 1959 | NGYWTNS |
| 3 | 432 | QDPHSKI |  | 53 | 1999 | PGTHRGL |
| 4 | 592 | ELKFNTY |  | 54 | 1999 | KTAVNGS |
| 5 | 604 | DPKIHVW |  | 55 | 1999 | MYPSHAN |
| 6 | 726 | SSDPHHT |  | 56 | 1999 | NNPAAAR |
| 7 | 870 | QTPEWGT |  | 57 | 1999 | TTRVADK |
| 8 | 989 | ENTPAGS |  | 58 | 1999 | YKATPNL |
| 9 | 1108 | LRETGMT |  | 59 | 1999 | NWPPRAD |
| 10 | 1214 | LHSVSAN |  | 60 | 2035 | HSDVPRL |
| 11 | 1214 | PNTIAKT |  | 61 | 821 | GRTDLAN |
| 12 | 1242 | NGDTMAT |  | 62 | 2068 | YTVPARY |
| 13 | 1258 | SWTDTRS |  | 63 | 2068 | HGEPFQS |
| 14 | 1302 | INGYEAR |  | 64 | 2068 | GYKYNYY |
| 15 | 227 | KYYQTTQ |  | 65 | 2068 | QPFNAQY |
| 16 | 1335 | MSTTTAF |  | 66 | 2068 | VGTTTAA |
| 17 | 1365 | LHAPLAI |  | 67 | 2068 | KHQSYEL |
| 18 | 1380 | THSTAKY |  | 68 | 2098 | HLHPGPS |
| 19 | 1380 | TFALNTN |  | 69 | 2098 | ELDRIRH |
| 20 | 1380 | GPNMKFV |  | 70 | 2098 | SMSPTLM |
| 21 | 1415 | MALNQSM |  | 71 | 2153 | TIHAANT |
| 22 | 1456 | NQNEHRL |  | 72 | 2153 | YAWSTRD |
| 23 | 1456 | TQDHMRI |  | 73 | 2153 | HGLLTRT |
| 24 | 1471 | TDSSTET |  | 74 | 2153 | EQKALKL |
| 25 | 1488 | SGAPNKY |  | 75 | 2153 | LTENAKN |
| 26 | 1488 | NHMKHAK |  | 76 | 2153 | LGSNQLA |
| 27 | 1525 | KATNLHL |  | 77 | 2193 | NLASHRY |
| 28 | 1558 | YHSSPPS |  | 78 | 2193 | DTSTKDY |
| 29 | 1558 | TGVATKV |  | 79 | 2193 | HTHLASV |
| 30 | 1573 | TNANMLI |  | 80 | 2234 | PNPPYTK |
| 31 | 1588 | PYSNVFH |  | 81 | 2234 | YGLMNAS |
| 32 | 1636 | TAAPWWN |  | 82 | 2234 | YGKESPT |
| 33 | 1674 | TMDKTKI |  | 83 | 2234 | APSSSYI |
| 34 | 1716 | DANNRME |  | 84 | 73 | TTLRVPL |
| 35 | 1754 | NTLSKHA |  | 85 | 2276 | DNGQLQM |
| 36 | 1773 | SSAYDTV |  | 86 | 2276 | NHWSLYL |
| 37 | 1794 | SLAHPRS |  | 87 | 2276 | MPSNNPI |
| 38 | 1794 | PSMGHHV |  | 88 | 2334 | SLNLFRD |
| 39 | 1794 | HQSANMY |  | 89 | 2334 | MWPSMKH |
| 40 | 1816 | FHQTHVA |  | 90 | 2334 | NTTLRTY |
| 41 | 1816 | FTGYHKH |  | 91 | 2334 | ASIHPVL |
| 42 | 1816 | NSLWTAL |  | 92 | 2387 | PVGDPRN |
| 43 | 1816 | TGSTWAN |  | 93 | 2387 | SDSLHRK |
| 44 | 1816 | TNWGNQI |  | 94 | 2445 | GHWHGTQ |
| 45 | 1848 | DPKAVNS |  | 95 | 2445 | TTGTPSV |
| 46 | 1848 | DVAPRKN |  | 96 | 2445 | NGHSEHH |
| 47 | 1880 | NDTNDLH |  | 97 | 2445 | NTTNYWS |
| 48 | 1880 | DHGHSNW |  | 98 | 2489 | PYTVSMM |
| 49 | 1919 | PANNHHM |  | 99 | 2489 | HTMVTTS |
| 50 | 1959 | QPTRTLQ |  | 100 | 2489 | NYGHTKD |

Un-R: unnormalized rank; N-R: normalized rank.

**Table S6. Downstream primers for the construction of plasmids expressing His-EGFP-C7C fusion proteins (pET14b/His-EGFP-C7C).**

| **No.** | **Primer name** | **Sequence** |
| --- | --- | --- |
| 1 | ELKFNTY | ATACGTATTAAACTTCAACTCACACTCGAGCTTGTACAGCTCGTCCATG |
| 2 | DPKIHVW | CCACACATGAATCTTAGGATCACACTCGAGCTTGTACAGCTCGTCCATG |
| 3 | NLIANRS | AGAACGATTCGCAATAAGATTACACTCGAGCTTGTACAGCTCGTCCATG |
| 4 | SSDPHHT | AGTATGATGCGGATCAGACGAACACTCGAGCTTGTACAGCTCGTCCATG |
| 5 | KSATPWT | AGTCCAAGGAGTAGCACTCTTACACTCGAGCTTGTACAGCTCGTCCATG |
| 6 | QTPEWGT | AGTACCCCACTCCGGAGTCTGACACTCGAGCTTGTACAGCTCGTCCATG |
| 7 | PSKTPTH | ATGAGTAGGCGTCTTAGACGGACACTCGAGCTTGTACAGCTCGTCCATG |
| 8 | KDSTHNN | ATTATTATGCGTCGAATCCTTACACTCGAGCTTGTACAGCTCGTCCATG |
| 9 | INSDPTR | ACGCGTAGGATCACTATTAATACACTCGAGCTTGTACAGCTCGTCCATG |
| 10 | TTLRVPL | CAAAGGCACACGCAGAGTAGTACACTCGAGCTTGTACAGCTCGTCCATG |
| 11 | GWSPPKM | CATCTTAGGCGGACTCCAACCACACTCGAGCTTGTACAGCTCGTCCATG |
| 12 | NMHTPMV | CACCATAGGCGTATGCATATTACACTCGAGCTTGTACAGCTCGTCCATG |
| 13 | QKSINLI | AATCAGATTAATACTCTTCTGACACTCGAGCTTGTACAGCTCGTCCATG |
| 14 | TWAPNHE | CTCATGATTAGGAGCCCACGTACACTCGAGCTTGTACAGCTCGTCCATG |
| 15 | LAYAHHT | CGTATGATGAGCATAAGCAAGACACTCGAGCTTGTACAGCTCGTCCATG |
| 16 | PLHPSTP | AGGCGTAGACGGATGAAGCGGACACTCGAGCTTGTACAGCTCGTCCATG |
| 17 | NKFSLAS | ACTAGCAAGCGAAAACTTATTACACTCGAGCTTGTACAGCTCGTCCATG |
| 18 | SENSPLL | CAGCAGAGGCGAATTCTCACTACACTCGAGCTTGTACAGCTCGTCCATG |
| 19 | KYYQTTQ | CTGCGTAGTCTGATAATACTTACACTCGAGCTTGTACAGCTCGTCCATG |
| 20 | VTKLGSL | CAGAGACCCAAGCTTAGTAACACACTCGAGCTTGTACAGCTCGTCCATG |
| 21 | QDPHSKI | AATCTTAGAATGAGGATCCTGACACTCGAGCTTGTACAGCTCGTCCATG |
| 22 | QTYHSGH | ATGACCACTATGATAAGTCTGACACTCGAGCTTGTACAGCTCGTCCATG |
| 23 | DAREKPS | AGAAGGCTTCTCCCTCGCATCACACTCGAGCTTGTACAGCTCGTCCATG |
| 24 | SYLNQKQ | CTGCTTCTGATTCAGATAACTACACTCGAGCTTGTACAGCTCGTCCATG |
| 25 | GLAAKDY | ATAATCCTTAGCCGCAAGACCACACTCGAGCTTGTACAGCTCGTCCATG |
| 26 | PANNHHM | CATATGATGATTATTCGCAGGACACTCGAGCTTGTACAGCTCGTCCATG |
| 27 | HMTHNIP | AGGAATATTATGAGTCATATGACACTCGAGCTTGTACAGCTCGTCCATG |
| 28 | SLNGLTS | AGACGTCAGACCATTCAGCGAACACTCGAGCTTGTACAGCTCGTCCATG |
| 29 | GINVQNY | ATAATTCTGCACATTAATCCCACACTCGAGCTTGTACAGCTCGTCCATG |
| 30 | HPDDRTV | CACCGTCCTATCATCCGGATGACACTCGAGCTTGTACAGCTCGTCCATG |
| 31 | SKTHVIH | ATGAATAACATGCGTCTTCGAACACTCGAGCTTGTACAGCTCGTCCATG |
| 32 | KSSTPTT | CGTAGTCGGCGTACTCGACTTACACTCGAGCTTGTACAGCTCGTCCATG |
| 33 | HNHPHIG | CCCAATATGAGGATGATTATGACACTCGAGCTTGTACAGCTCGTCCATG |
| 34 | IHSPTAL | CAGCGCAGTAGGCGAATGAATACACTCGAGCTTGTACAGCTCGTCCATG |
| 35 | NAGHLSQ | CTGAGACAGATGACCCGCATTACACTCGAGCTTGTACAGCTCGTCCATG |
| 36 | NIKSSHV | AACATGACTACTCTTAATATTACACTCGAGCTTGTACAGCTCGTCCATG |
| 37 | ISSSINH | ATGATTAATCGAACTAGAAATACACTCGAGCTTGTACAGCTCGTCCATG |
| 38 | LTAKHMQ | CTGCATATGCTTAGCAGTAAGACACTCGAGCTTGTACAGCTCGTCCATG |
| 39 | FATRADH | ATGATCAGCACGAGTCGCAAAACACTCGAGCTTGTACAGCTCGTCCATG |
| 40 | SAQQPAS | ACTAGCCGGCTGCTGCGCCGAACACTCGAGCTTGTACAGCTCGTCCATG |
| 41 | PKGDENT | CGTATTCTCATCACCCTTCGGACACTCGAGCTTGTACAGCTCGTCCATG |
| 42 | PSTVPWS | ACTCCACGGCACCGTCGACGGACACTCGAGCTTGTACAGCTCGTCCATG |
| 43 | SKEATPF | AAAAGGCGTCGCCTCCTTACTACACTCGAGCTTGTACAGCTCGTCCATG |
| 44 | LNRYVAD | ATCCGCAACATACCGATTCAAACACTCGAGCTTGTACAGCTCGTCCATG |
| 45 | MNQASMS | ACTCATAGAAGCCTGATTCATACACTCGAGCTTGTACAGCTCGTCCATG |
| 46 | GGSMLSS | AGACGACAGCATACTACCCCCACACTCGAGCTTGTACAGCTCGTCCATG |
| 47 | HPVWPTY | ATACGTCGGCCACACAGGATGACACTCGAGCTTGTACAGCTCGTCCATG |
| 48 | SWGINVT | AGTCACATTAATACCCCAACTACACTCGAGCTTGTACAGCTCGTCCATG |
| 49 | TSNADNT | CGTATTATCCGCATTACTCGTACACTCGAGCTTGTACAGCTCGTCCATG |
| 50 | NEIARQY | ATACTGCCTCGCAATCTCATTACACTCGAGCTTGTACAGCTCGTCCATG |
| 51 | SVWLMNK | CTTATTCATCAGCCAAACCGAACACTCGAGCTTGTACAGCTCGTCCATG |
| 52 | QYGFHSR | CCGCGAATGAAACCCATACTGACACTCGAGCTTGTACAGCTCGTCCATG |
| 53 | YTNSQTN | ATTCGTCTGACTATTAGTATAACACTCGAGCTTGTACAGCTCGTCCATG |

**Table S7. Amino acid sequences and physical features of synthetic peptides.**

| **No.** | **Peptide name** | **Peptide sequence** | **MW** | **Net charge** | **PI** | **GRAVY** |
| --- | --- | --- | --- | --- | --- | --- |
| **1** | **VTK** | **CVTKLGSLC** | **923.15** | **0.8** | **8.23** | **1.222** |
| **2** | **KYY** | **CKYYQTTQC** | **1137.29** | **0.8** | **8.21** | **-1.1** |
| **3** | **ISS** | **CISSSINHC** | **963.09** | **0.1** | **7.25** | **0.544** |
| **4** | **INS** | **CINSDPTRC** | **1008.13** | **-0.1** | **6.06** | **-0.567** |
| **5** | **LL37** | **LLGDFFRKSKEKIGKEFKRIVQRIKDFLRNLVPRTES** | **4493.32** | **5.9** | **11.35** | **-0.7243** |
| **6** | **VTK-LL37** | **CVTKLGSLCVPGVGLLGDFFRKSKEKIGKEFKRIVQRIKDFLRNLVPRTES** | **5807.95** | **6.9** | **10.90** | **-0.1922** |
| **7** | **LL37-VTK** | **LLGDFFRKSKEKIGKEFKRIVQRIKDFLRNLVPRTESVPGVGCVTKLGSLC** | **5807.95** | **6.9** | **10.90** | **-0.1922** |
| **8** | **KYY-LL37** | **CKYYQTTQCVPGVGLLGDFFRKSKEKIGKEFKRIVQRIKDFLRNLVPRTES** | **6022.09** | **6.9** | **10.55** | **-0.6020** |
| **9** | **LL37-KYY** | **LLGDFFRKSKEKIGKEFKRIVQRIKDFLRNLVPRTESVPGVGCKYYQTTQC** | **6022.09** | **6.9** | **10.55** | **-0.6020** |
| **10** | **ISS-LL37** | **CISSSINHCVPGVGLLGDFFRKSKEKIGKEFKRIVQRIKDFLRNLVPRTES** | **5847.89** | **6.1** | **10.78** | **-0.3118** |
| **11** | **INS-LL37** | **CINSDPTRCVPGVGLLGDFFRKSKEKIGKEFKRIVQRIKDFLRNLVPRTES** | **5892.93** | **5.9** | **10.77** | **-0.5078** |
